# Sea Star Wasting Disease in the Keystone Predator *Pisaster ochraceus* in Oregon: Insights into Differential Population Impacts, Recovery, Predation Rate, and Temperature Effects from Long-Term Research

**DOI:** 10.1371/journal.pone.0153994

**Published:** 2016-05-04

**Authors:** Bruce A. Menge, Elizabeth B. Cerny-Chipman, Angela Johnson, Jenna Sullivan, Sarah Gravem, Francis Chan

**Affiliations:** Department of Integrative Biology, Oregon State University, Corvallis, Oregon, United States of America; The Evergreen State College, UNITED STATES

## Abstract

Sea star wasting disease (SSWD) first appeared in Oregon in April 2014, and by June had spread to most of the coast. Although delayed compared to areas to the north and south, SSWD was initially most intense in north and central Oregon and spread southward. Up to 90% of individuals showed signs of disease from June-August 2014. In rocky intertidal habitats, populations of the dominant sea star *Pisaster ochraceus* were rapidly depleted, with magnitudes of decline in density among sites ranging from -2x to -9x (59 to 84%) and of biomass from -2.6x to -15.8x (60 to 90%) by September 2014. The frequency of symptomatic individuals declined over winter and persisted at a low rate through the spring and summer 2015 (~5–15%, at most sites) and into fall 2015. Disease expression included six symptoms: initially with twisting arms, then deflation and/or lesions, lost arms, losing grip on substrate, and final disintegration. SSWD was disproportionally higher in orange individuals, and higher in tidepools. Although historically *P*. *ochraceus* recruitment has been low, from fall 2014 to spring 2015 an unprecedented surge of sea star recruitment occurred at all sites, ranging from ~7x to 300x greater than in 2014. The loss of adult and juvenile individuals in 2014 led to a dramatic decline in predation rate on mussels compared to the previous two decades. A proximate cause of wasting was likely the “Sea Star associated Densovirus” (SSaDV), but the ultimate factors triggering the epidemic, if any, remain unclear. Although warm temperature has been proposed as a possible trigger, SSWD in Oregon populations increased with cool temperatures. Since *P*. *ochraceus* is a keystone predator that can strongly influence the biodiversity and community structure of the intertidal community, major community-level responses to the disease are expected. However, predicting the specific impacts and time course of change across west coast meta-communities is difficult, suggesting the need for detailed coast-wide investigation of the effects of this outbreak.

## Introduction

The outbreak of Sea Star Wasting Disease (SSWD) that began in summer 2013 along the West Coast of North America was one of the largest epidemics in a marine ecosystem in recorded history [1–3], rivaling well-known disease outbreaks in terrestrial systems [4–6]. It affected 20+ species of sea stars ranging from Baja California, Mexico to Alaska, USA and caused severe declines in sea star populations throughout the affected region (http://www.seastarwasting.org). The grotesque manner in which sea stars “melt” (figure 1 in [3], see also Figs A-H in S1 Appendix) as a result of the disease has generated scientific and public concern over the fate of these iconic marine species. In addition to seizing public attention, SSWD provides a unique scientific opportunity to investigate several important ecological questions. First, studying the time course and intensity of the outbreak informs our understanding of disease outbreaks in marine systems, which are predicted to increase with climate change and increasing anthropogenic impacts (e.g., [7]). Second, recovery of sea star populations after the disease will provide unprecedented insight into connections between meta-populations that exchange planktonic larvae. Finally and most importantly, SSWD has not only impacted sea star populations, but is expected to cause drastic shifts in marine communities. In particular, several foundational ecological concepts, including keystone predation, the intermediate predation hypothesis, trophic cascades, and indirect effects were originally based on experiments with the intertidal keystone predator *Pisaster ochraceus* [8–10], one of the species devastated by the disease. Because SSWD has led to massive declines in *P*. *ochraceus*, this event provides an unprecedented opportunity to revisit the generality of these fundamental concepts over space and time.

We believe that, combined with research reported in other publications ([1–3,11], the study of SSWD exemplifies the approach advocated by Burge et al. [12]: a “union of the modern and the classic” approaches to understanding marine diseases. For several reasons, the impact of disease on populations and communities is difficult to investigate [3]. When effects are sublethal, for example, identifying disease as a cause of reduced performance (e.g., growth, reproduction) or a factor underlying mortality and thus population size is generally more difficult than detecting the effects of other species interactions, such as competition, predation or facilitation. Even when lethal, epidemics can be particularly difficult to discern in aquatic habitats, especially in the absence of long-term observations [12]. Often, disease epidemics occur so fast that ecologists and microbiologists have limited opportunities to investigate, making pathogen identification, culturing, and determination of specific impacts under controlled situations very difficult if not impossible. Since some hypothesize that diversity, expression, and prevalence of diseases will increase in the future as the climate warms and anthropogenic influences on environments increase ([7,13,14], but see [15]), ecological investigations of diseases and their impacts are increasingly needed if we are to forecast and prepare for possible future ecosystem changes.

Sea stars are diverse and ubiquitous in the world’s oceans [16,17], and some sea star species are known to have been susceptible to “sea star wasting disease” in the past [3]. These outbreaks, however, have been brief and localized, inhibiting intensive investigation. For example, Dungan et al. [18] reported “scores of disintegrating sun stars” during an outbreak in the rocky intertidal sun star *Heliaster kubinjii* in the Gulf of California in 1978. Unfortunately, the sun star was virtually extinct within three years, and the cause of the disease was never identified. Similar SSWD outbreaks in sea stars were reported at about the same time (late 1970’s; References and Notes in [18,19]), but these too were usually brief (months) and localized. Later reports of SSWD were made in southern California coincident with El Niño-Southern Oscillation (ENSO) events [20,21]. Since these outbreaks were localized, affected few species, and were relatively brief, pathogens involved were not investigated. In contrast to these previous epidemics, the 2013–15 SSWD event was unprecedented in scale and duration. Because it also occurred in a system we know well, the current outbreak provides an ideal opportunity to identify the large-scale ecological impacts of disease.

### The 2013–2015 Epidemic

We have been studying sites along 360 km of the Oregon coast for 15–32 years. A main focus of our research has been examination of community structure and how it varies spatially and temporally in relation to climatological, oceanographic, and other environmental conditions. Thus, we have a detailed historical understanding of this system and knowledge of typical patterns of variation.

Eisenlord et al. (3) reported results from a study similar to ours in Washington state. On the basis of modeling and comparisons of temperatures to SSWD, and laboratory experiments monitoring mortality rate of infected sea stars under different temperatures, they suggested that warm temperatures triggered the outbreak, at least in the San Juan Islands. Evidence for a thermal effect from southern Puget Sound and one outer coast site was inconclusive.

**Fig 1:**
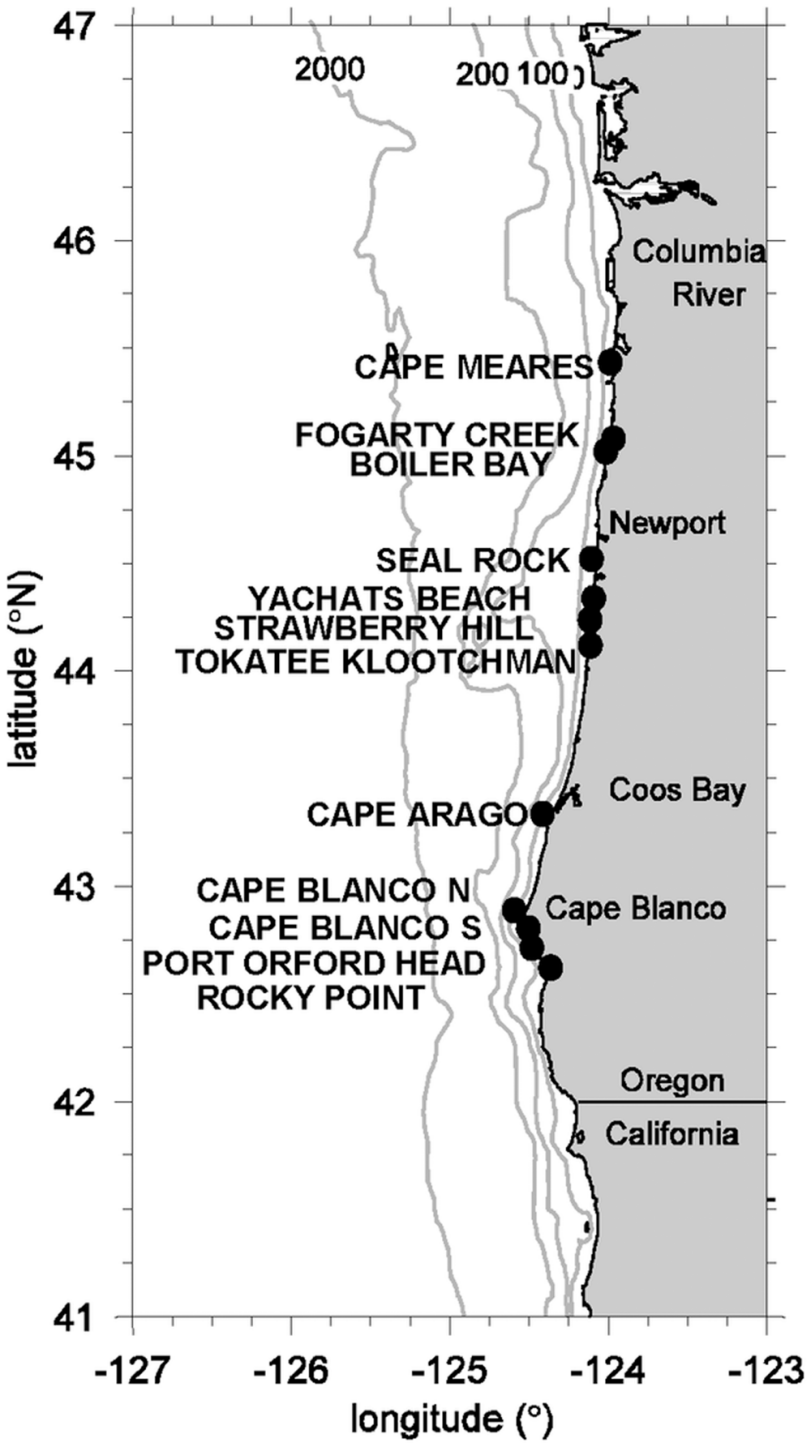
Map of study sites along the Oregon coast. Subtidal bathymetry is shown with gray lines, and scaled in m depth.

In spring 2013, after reports that massive sea star die-offs caused by SSWD had been observed subtidally off Vancouver BC and in southern and central California, we intensified our ongoing observations on *P*. *ochraceus* at our 12 long-term study sites along the Oregon coast (Fig 1). Despite the accelerating reports of SSWD in other regions, we saw no convincing evidence of the disease in *P*. *ochraceus* populations through March 2014. In April 2014, however, while sampling *P*. *ochraceus* populations for wet mass, arm length, feeding, and condition, we encountered a few possibly-diseased sea stars at one site. This observation triggered the initiation of intensive quantitative surveys of *P*. *ochraceus* populations at all sites. Below, we report the results of surveys assessing the intensity, symptoms, time-course, and population impacts of the disease on *P*. *ochraceus*, including an investigation of susceptibility of individuals by age, subhabitat and sea star color. In addition, we detail an unprecedented influx of small recruits of *P*. *ochraceus*, examine the potential causes, and explore whether these recruits will lead to the recovery of the sea star population. We also show that the demise of this ecologically important predator has led to decreased predation on their mussel prey, and speculate on the potential ramifications for the ecosystem. Finally, we examine the relationship between wasting frequency and environmental conditions, including temperature and ocean acidification, finding that SSWD frequency was associated with cool, not warm temperatures.

## Study Sites and Methods

### Ethics Statement

Although no animals were collected for this research, the Oregon Department of Fish and Wildlife requires collecting permits for all coastal researchers. Ours were permit numbers 18510 (2014) and 19272 (2015). All site access was public except for Fogarty Creek, whose owners have granted us indefinite access to the site. No protected species were sampled.

### Field Surveys

After finding SSWD at all 12 study sites in April 2014 (Fig 1), we focused our intensive surveys on the 9 core sites (all those in Fig 1 except Cape Meares, Seal Rock, and Cape Arago) with the most extensive sets of supplementary observations and experiments. Sites were nested within capes (= regions), with (from north to south; see Fig 1) Fogarty Creek (FC) and Boiler Bay (BB) occurring within Cape Foulweather (CF); Yachats Beach (YB), Strawberry Hill (SH), and Tokatee Klootchman (TK) occurring within Cape Perpetua (CP), and Cape Blanco North (CBN), Cape Blanco South (CBS), Port Orford Heads (POH), and Rocky Point (RP) occurring within Cape Blanco (CB). All sites had *P*. *ochraceus* populations, and typical patterns of community structure (see [22,23] for details). Hereafter we refer to site and capes by the codes listed above.

*Wasting Surveys*. We conducted two types of surveys to assess disease patterns: wasting (WS) and size and density surveys (SDS). WS involved observing and classifying the status of all sea stars we could find during low tides. Each sea star was classified as showing disease symptoms or not (“healthy” vs. “sick” or “wasted”). Individuals were then separated into color groups (purple vs. orange), subhabitats (in or out of tidepools), and size/age groups (adults and juveniles; and in 2015, recruits). Those with disease symptoms were categorized in order of increasing severity as: (1) having twisted arms, (2–3) lesions and/or being deflated, (4) having recently lost arms, (5) losing their grip on the substratum, or (6) dissolving or disintegrating or “melting” (see [3], Figs A-H in S1 Appendix for photos). These surveys were repeated at the core sites as often as possible, ranging in frequency from every few days to two weeks (spring and summer 2014, 2015) to months (fall 2014) apart. We conducted a total of 147 surveys from 18 April 2014 to 31 October 2015. We avoided touching animals due to uncertainty about the mechanisms of transmission of the disease. Thus, most observations were of symptoms on the aboral surface (occasional observations revealed no symptoms on oral surfaces), and animals deep in crevices or other inaccessible habitats could not be categorized. In 2014, we kept track of whether individuals were “adults” (>70g or about 15 cm in diameter) or “juveniles” (10 to 70 g or about 5–15 cm in diameter; see [3,24]). In 2015 surveys we added “recruits” (individuals <10g and <5 cm in diameter) because of a large pulse of small sea stars appearing at many sites between fall 2014 and spring 2015.

We counted sea stars as orange only when they were a distinctive orange hue. All others, including shades of purple, brown, maroon or blue were categorized as purple. New recruits usually began as white or gray (< 2cm diameter) with aboral radial lines of bluish tint. We arbitrarily categorized these as “purple” for calculation of wasting frequencies. Color differentiation developed with growth; most recruits were bluish at first (1–3 cm diameter) and then gradually became mottled hues of purple, brown and orange (2–5 cm diameter).

*Size and Density Surveys*. In spring and summer we used replicated “belt” transects (n = 5/site) to quantify the sizes of individuals (“standard” arm length, defined as the distance from the madreporite to the tip of the opposite arm) and density (number per m^2^). Each transect spanned 5-10m horizontally and 2m vertically in the low zone just below the mussel bed. In each, we counted and measured all sea stars, and before the disease (pre-2014) we weighed each sea star. During the 2014 outbreak, we did not directly weigh individuals to avoid serving as agents of disease transmission. Instead, we used site-specific surveys from summer 2013 at each site to generate linear regressions between arm length and wet mass, and used these to convert arm lengths measured in 2014 to estimated wet mass for each individual. We resumed weighing sea stars in summer 2015 after SSWD frequencies had declined to mostly single digits.

### Settlement

The life cycle of *P*. *ochraceus’* is as follows. Energy from food consumed during summer is initially stored in pyloric caecae, then during winter, is transferred to the gonads [25–27]. Mass spawning occurs in spring (in Oregon, typically in May; [26]), after which the cycle begins anew. Each female produces millions of eggs, which are fertilized in the water column, and develop into planktotrophic bipinnaria larvae (e.g., [28]). Development to the settling brachiolaria larval stage takes ~4–6 weeks and settlement starts in July, continuing into the fall. In Oregon, surviving recruits (5–15 mm in diameter) are first reliably detectable starting in April the following spring, when tides switch to daytime and wave action begins to decrease.

We used two methods to quantify the input of new settlers and recruits (individuals < 10g and < 5 cm in diameter or ~2.5 cm in standard arm length) into populations on the shore. To estimate sea star settlement (individuals ~1 mm diameter and ≤ 1 month after settlement), we deployed “turfies,” which were squares of astroturf (turf dimensions = 15 x 15 cm; n = 5 per site; e.g., [29]) deployed into the low intertidal at four sites, FC, BB, YB and SH. Sampling began in 2002; in 2013 we added RP and CBN sites. Turfies were held against a PVC basal plate that was fastened to the rock using lag screws with eight wing nuts and machine screws around the edges of the plate. Turfies were deployed in May and exchanged monthly through September or October. Collected turfies were taken to the lab and frozen at -20°C, then processed by shaking each thawed turfy in a plastic dishpan of water until all material had been extracted. The contents of the dishpan were poured through a 417 μm sieve and examined under a microscope to obtain counts of recently settled *P*. *ochraceus*, crabs, sea urchins and *Leptasterias* spp. The latter were distinguishable from *P*. *ochraceus* settlers by having six instead of five arms and usually being larger. Though settlement into turfies does not necessarily reflect settlement onto natural surfaces (algae, rock, mussels, barnacles, etc.), and settlers could either settle or crawl onto turfies, they provide a standardized surface for comparison within and among sites.

### Recruitment

Quantification of *P*. *ochraceus* recruits used the SDS data, in which larger/older recruits ≥ 10 mm in diameter were reliably detectable. Based on inspection of spring and summer surveys each year, and judging approximate growth from inspection of size frequency distributions, we judged that “recruits’ are animals < 3 cm in diameter and are from the previous year’s cohort of settlers. The presumed age of most of these individuals is about 6–9 months, depending on when the transect survey was done in the spring/summer following settlement in the previous late summer or fall.

### Predation Rate Experiments

Predation rates on *M*. *californianus* by *P*. *ochraceus* have been quantified periodically at two sites, BB and SH, since 1990 ([23,30]), and annually since 2007 at four additional sites (FC, YB/TK, CBN, and POH). Population predation rates were assessed by transplanting clumps of mid-intertidal zone mussels of 4–6 cm in shell length to pairs (n = 5) of prepared plots, holding them against the rock substratum using Vexar ^®^ mesh covers fastened to the substrate using lag screws inserted into holes with wall anchors, and allowing them to reattach for 4–6 weeks [23,30]. After mesh removal, we fastened either complete (-*Pisaster*) or partial stainless steel mesh fences (+*Pisaster*) around plots. Treatments were assigned by coin flip. Thereafter, the number of mussels surviving in each plot were counted every two (summer, early fall) to four (fall, winter) weeks. Experiments at a site were ended when all mussels had been eaten in +*Pisaster* plots, or in the following May, when new experiments were begun.

Virtually all predation in these plots was due to *P*. *ochraceus*. The mussels used were too large for the whelk *Nucella ostrina* and the small six-armed sea star *Leptasterias* spp. to consume, the whelk *N*. *canaliculata* will not eat *M*. *californianus* in Oregon [31–34], and crabs *Cancer productus* were uncommon at all of our sites.

### Temperature

To examine the possible role of temperature, we analyzed 2014 data from a long-term intertidal thermal sensor array, which included 3–6 replicate sensors (a mix of HOBO Temperature/Light Data Logger, Onset Computer Corp. UA-002-64, and HOBO TidbiT v2 Temp Data Logger, Onset Computer Corp. UTBI-001) deployed in the low intertidal zone at each site. Loggers recorded data every two minutes. To protect them from damage, sensors were housed in stainless steel mesh cages held down by lag screws. We present data from three sites, one each on CF (FC), CP (SH), and CB (CBN) and compare 2014 data to the long-term “climatology;” i.e., the long-term mean temperature plus 1 SD “envelopes” which enable detection of anomalous means.

### pH

Intertidal pH was measured using custom-designed Durafet^®^-based (Honeywell Inc.) pH sensors [35]. At SH, sensors were secured to the bedrock during low tides at -0.31 m below Mean Lower Low Water, and on a mooring in 15 m depth (4 m sensor depth) just offshore. Sensors were serviced at approximately 4-week intervals. As with intertidal temperature records, data taken when sensors were out of water during low tides were separated from in-water temperatures using tidal height time-series and surveyed height of the sensors. Data spikes (outliers) were filtered out using a ±4 standard deviation window. Each unit was calibrated directly against seawater and/or TRIS-based certified reference material (CRM) from A. Dickson’s group (Scripps Institute of Oceanography) or indirectly against seawater CRM-calibrated spectrophotometrically-determined pH samples. Thus, each deployment record was traceable to a CRM standard. Temperature was also recorded from each pH sensor and calibrated against a manufacturer-calibrated Seabird Electronics (SBE) thermograph in the laboratory. Mooring data were collected 15 April to 22 September 2014 and intertidal data were collected 16 May to 10 August 2014.

### Data Analysis

Data were analyzed using JMP PRO 12.0.1 (SAS Institute Inc., 2015; http://www.jmp.com). For analysis, all data except temperatures were normalized using either arcsine- (percent; arcsin[sqrt[x*0.01]]) or ln- (counts, densities; ln [x+1]) transformations. In all cases, we examined residuals visually to evaluate the assumption of normality and homoscedasticity. Leverage plots were inspected to identify outliers. We used analysis of variance and/or regression approaches in most data analyses. Linear contrasts were used for post-hoc comparisons. Linear regression analyses evaluated how sea star density changed during the SSWD epidemic. Nested 3-way ANOVA analyzed the decline in biomass between seasons (spring, summer) and years (2014, 2015) by cape and site nested within cape, and the effects of cape, zone, year, month nested within year and site nested within cape on *P*. *ochraceus* settlement. Predation rate experiments were analyzed with nested 2-way ANOVA, with factors cape, site nested within cape, and “era” (prewasting vs. wasting). Multiple linear regression was used to determine the relationships among SSWD frequency, pH and temperature (both averaged over the previous week, the previous two weeks and the previous month) at Strawberry Hill. Akaike Information Criterion corrected for finite sample sizes (AICc) was used to determine the best fit model.

## Results

### Wasting Surveys (WS)

**Fig 2:**
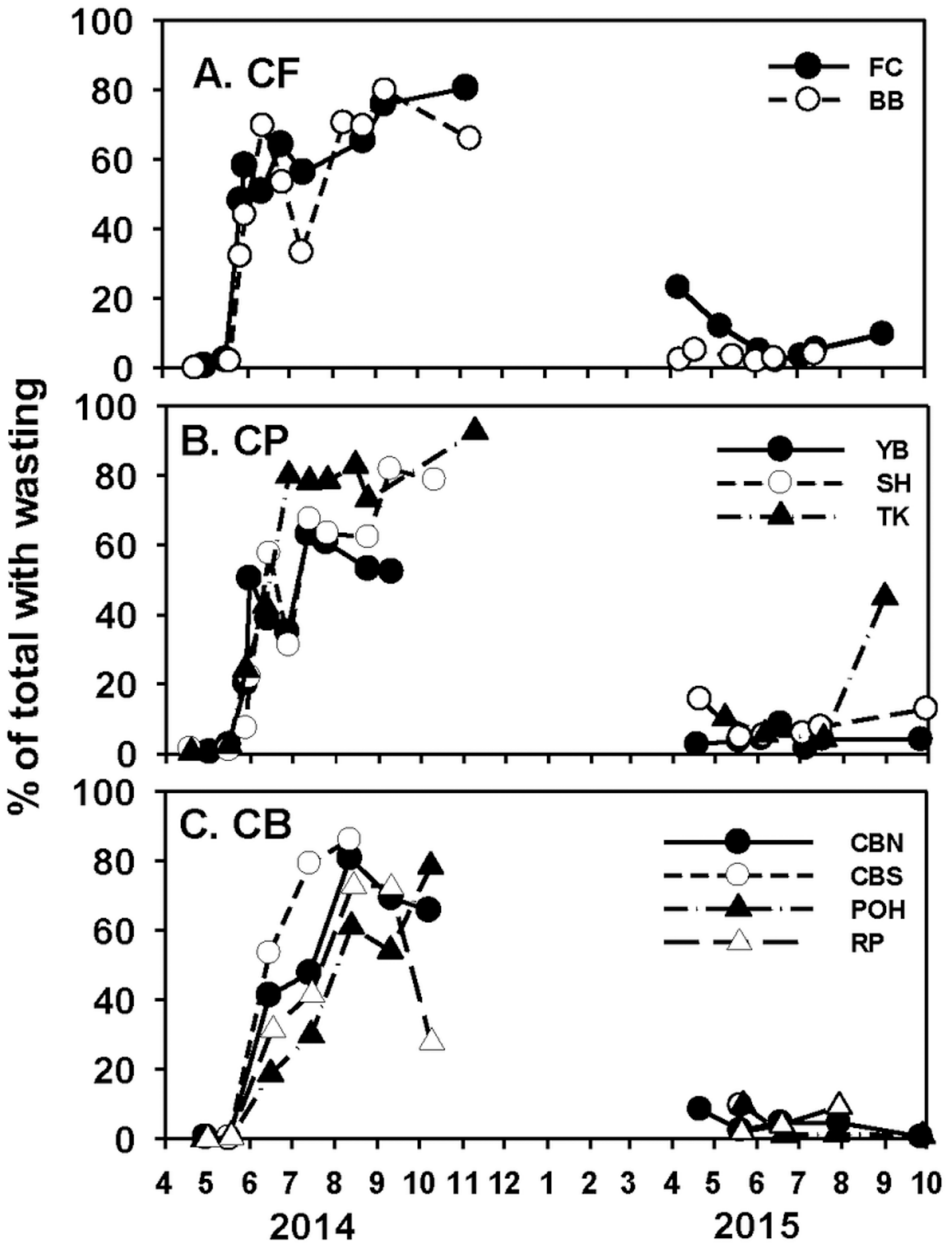
Wasting frequency at nine (eight in 2015) sites spread across three capes in 2014 and 2015. A. and D., CF = Cape Foulweather; B. and E., CP = Cape Perpetua; and C. and F., CB = Cape Blanco. Note that the y-axis scale varies among capes for 2015 data. See Fig 1 for site names, here coded as initials.

A total of 70,198 *P*. *ochraceus* observations were recorded across all sites from April 2014 to July 2015. By late April 2014, SSWD had infected populations at all our sites, but at low rates (<1%, Fig 2A–2C). Rates were still low (3–5%) two weeks later, but by the end of May 2014, infection frequencies had accelerated at the two most northerly sites, FC and BB. By the end of June, rates were all >40% at all but the two most southerly sites (Fig 2A–2C; CF = 52.3 ± 3.7%, CP = 39.9 ± 6.1%, CB = 18.4 ± 7.6%; 1-way ANOVA on late May/June data; F_2,27_ = 8.11, p = 0.0017, n = 30; adj R^2^ = 0.329). Interestingly, although SSWD frequencies increased exponentially from late May into June, rates of increase were faster to the north than to the south (Fig 2A–2C).

By mid/late summer 2014, rates of SSWD were all in the 60% to 80% range and remained high through the end of the year, at least at those sites where weather conditions allowed observations. By spring 2015, wasting had declined to 25% or less at the 8 sites still being monitored (Fig 2D–2F). Frequencies were low in summer 2015 (mostly < 10%) and stayed low into September 2015 with the exception of TK, where the last sample taken had risen sharply to 45% (Fig 2E). A similar, but unquantified September outbreak was observed at SH (S. Gravem, pers. obs.).

**Fig 3:**
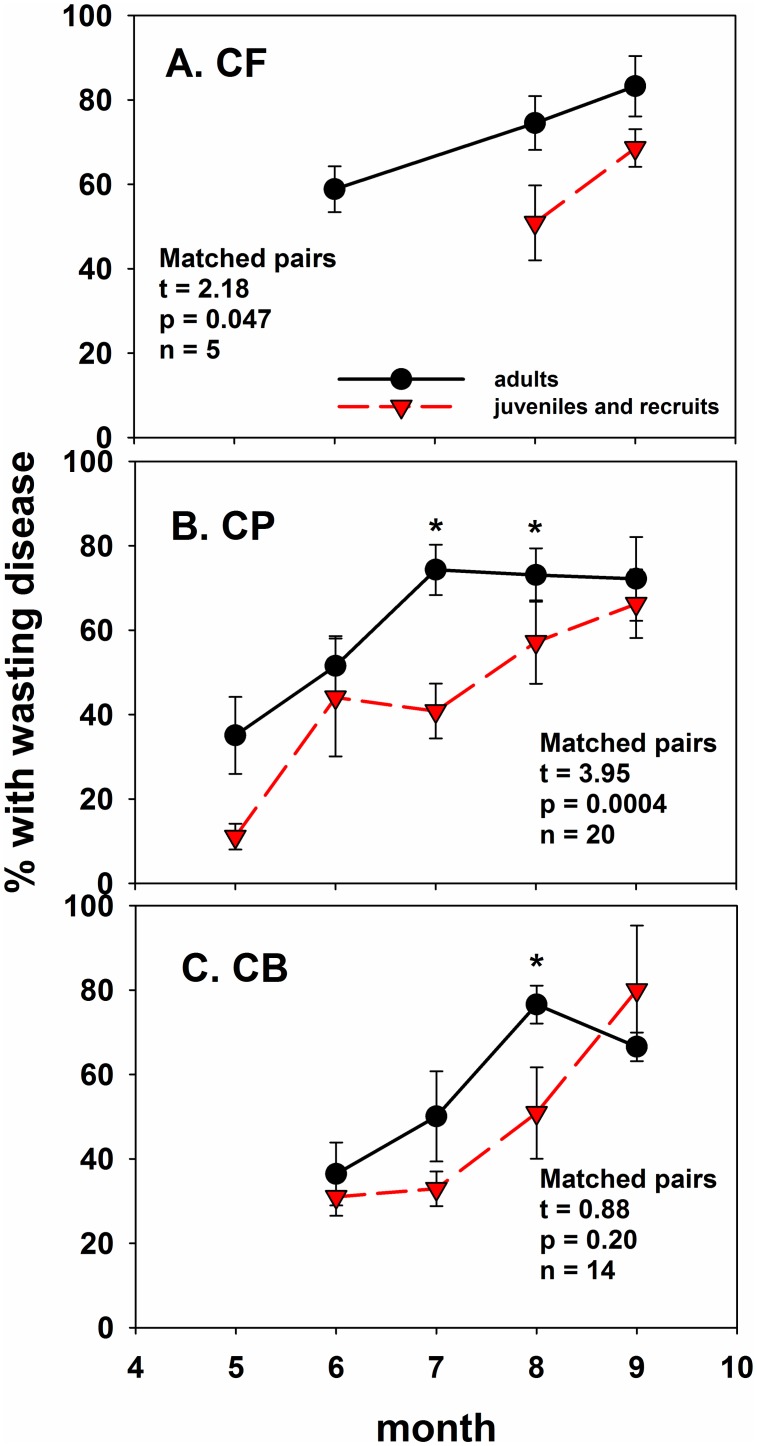
Frequency of small/juvenile *P*. *ochraceus* with wasting compared to frequency of adult *P*. *ochraceus* with wasting, averaged across sites within capes (± 1 SE). Asterisks indicate months in 2014 in which juveniles had lower % wasting, and matched pairs statistics indicate overall differences across months within capes. Data were sine^-1^ square root-transformed for analysis. If September data are dropped for CB, t = 3.33, p = 0.004, n = 11.

Through most of the summer, and consistent with (3), juvenile sea stars had lower SSWD frequencies than adults (Fig 3). Although monthly frequencies differed only at CP (July and August) and CB sites (August; Fig 3B and 3C), cape-scale percent SSWD June-August 2014 averages were consistently lower in juveniles vs. adults at CF and CP, but not at CB (Fig 3; matched pairs t-tests). At CB, juvenile SSWD frequency was lower than adults as well if the September sample was dropped (Fig 3C, see caption). By September, SSWD frequencies in juveniles and recruits approached or exceeded those of adults, perhaps because few adults remained at those sites.

In the early summer, adult sea stars of different colors and sub-habitats were differentially susceptible to SSWD (Table 1). Purple sea stars made up ~80% and orange individuals 20% of all populations, and sea stars tended to be found more often outside (~73%) than inside (~27%) tidepools. If sea stars across color and sub-habitat combinations were equally affected with wasting, the proportion of each color or sub-habitat among asymptomatic (“normal”) and symptomatic (“wasting”) should be similar. Early in the April-November field season, purple sea stars outside tidepool were infected less (43.6 ± 6.2% in May) than normal purple sea stars (60.0 ± 5.0%). This difference persisted through July (Table 1). Similarly, in early summer SSWD animals of both colors in tidepools were infected more frequently than normal animals (Table 1).

**Table 1:** Comparison of percentages of total number of *P*. *ochraceus* that were “normal” or “wasting” (shown in paired columns) in all combinations of color and subhabitat by month, summer 2014^a^.

Month (2014)	Normal Orange Out	Wasting Orange Out	Normal Purple Out	Wasting Purple Out	Normal Orange Pool	Wasting Orange Pool	Normal Purple Pool	Wasting Purple Pool	N
May	12.1 ± 0.7	12.7 ± 2.6	**60.0 ± 5.0**	**43.6 ± 6.2**	**6.1 ± 1.1**	**11.4 ± 2.3**	**21.8 ± 4.4**	**32.6 ± 6.5**	7
June	14.5 ± 1.9	15.3 ± 1.7	**63.0 ± 3.4**	**53.7 ± 3.1**	7.5 ± 2.6	5.7 ± 1.0	**15.7 ± 2.5**	**25.1 ± 3.5**	13
July	15.2 ± 1.9	17.3 ± 1.8	**68.7 ± 2.1**	**60.9 ± 2.1**	4.7 ± 1.0	6.3 ± 2.0	11.4 ± 1.2	15.5 ± 2.6	11
August	19.9 ± 2.3	19.5 ± 1.7	69.1 ± 3.1	70.0 ± 2.0	2.7 ± 0.7	3.2 ± 0.9	8.4 ± 1.5	7.3 ± 2.0	11
September	15.8 ± 1.6	17.6 ± 1.7	70.0 ± 4.4	73.3 ± 3.1	4.3 ± 1.0	3.3 ± 1.2	9.8 ± 2.9	6.0 ± 1.7	7

^a^Month pairs that were different and changed across months through the summer (tested with linear contrasts on months after two-way anova on difference between wasting and normal arcsine-transformed percentages by cape and month; cape was never significant) are in boldface.

Late in the 2014 field season, SSWD frequencies had increased in all categories (Fig 2), and disparities among the frequencies of SSWD and asymptomatic sea stars of different colors and subhabitats had disappeared (Table 1). As suggested by the changing proportions in successive months in Table 1, these changes seem likely due to the declines in numbers of differentially susceptible sea stars.

With the exception of RP, lesions were the most frequent symptoms observed across the nine populations (Fig I in S1 Appendix). At RP, animals with twisting, losing grip and deflation were almost as common as animals with lesions. Twisted animals tended to be second most common, while the frequencies of other symptoms varied among sites in their occurrence.

**Fig 4:**
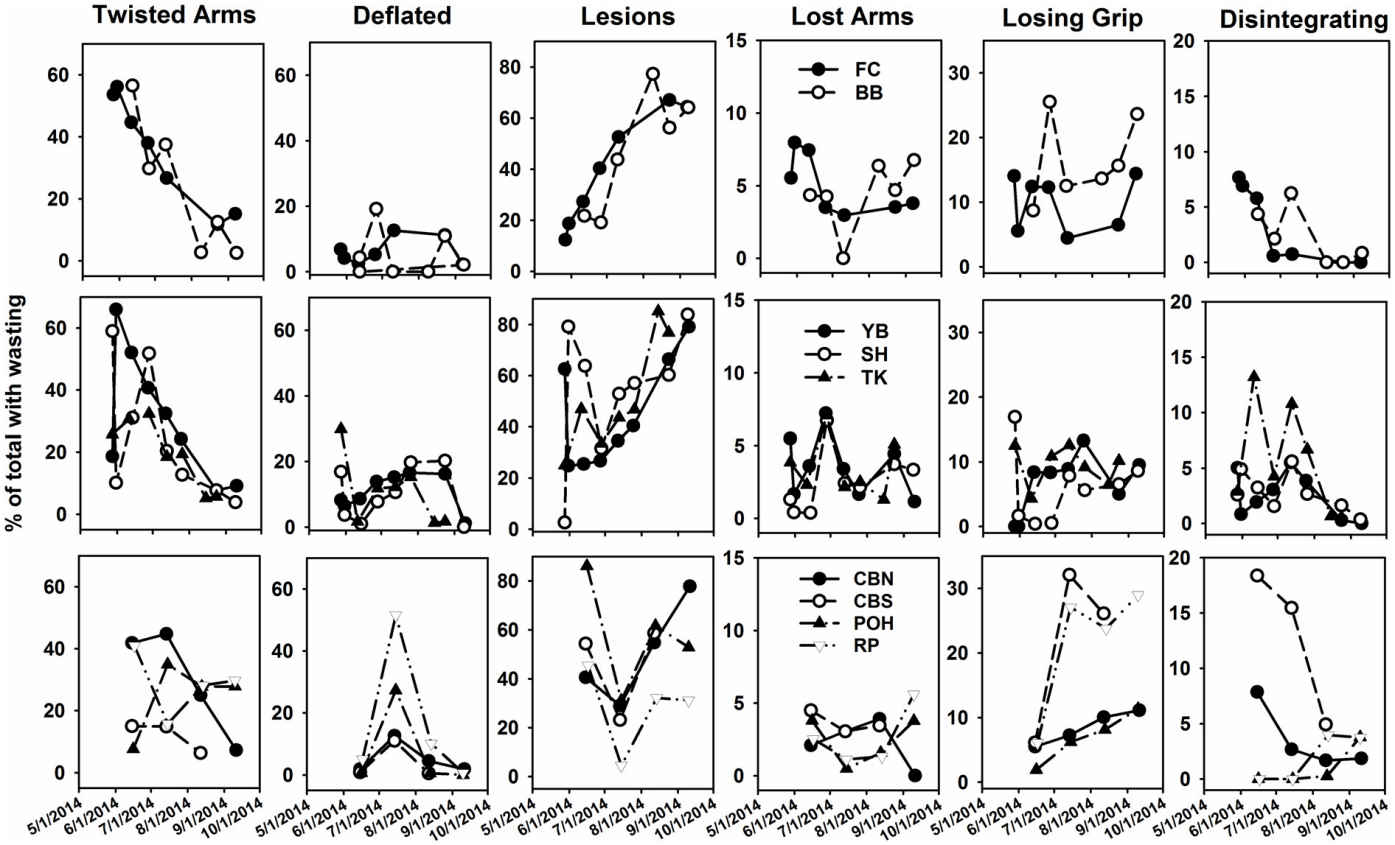
Frequencies of sea star wasting symptoms for *P*. *ochraceus* through time, summer 2014, at 9 sites. Top row = Cape Foulweather sites, middle row = Cape Perpetua sites, and bottom row = Cape Blanco sites. See Fig 1 caption for site names.

Through time across sites and capes, twisting was initially the most common symptom, declining as other symptoms became more common (Fig 4). Lesion frequency was generally initially low, but increasing at CF and CP sites. At CB, lesion frequencies were more variable, being high except for a sharp drop in mid-July with a rebound in August. Other symptoms varied through the summer, with lost arms tending to increase and deflation and disintegration tending to decrease toward summer’s end (Fig 4).

### Size and Density Surveys (SDS)

**Fig 5:**
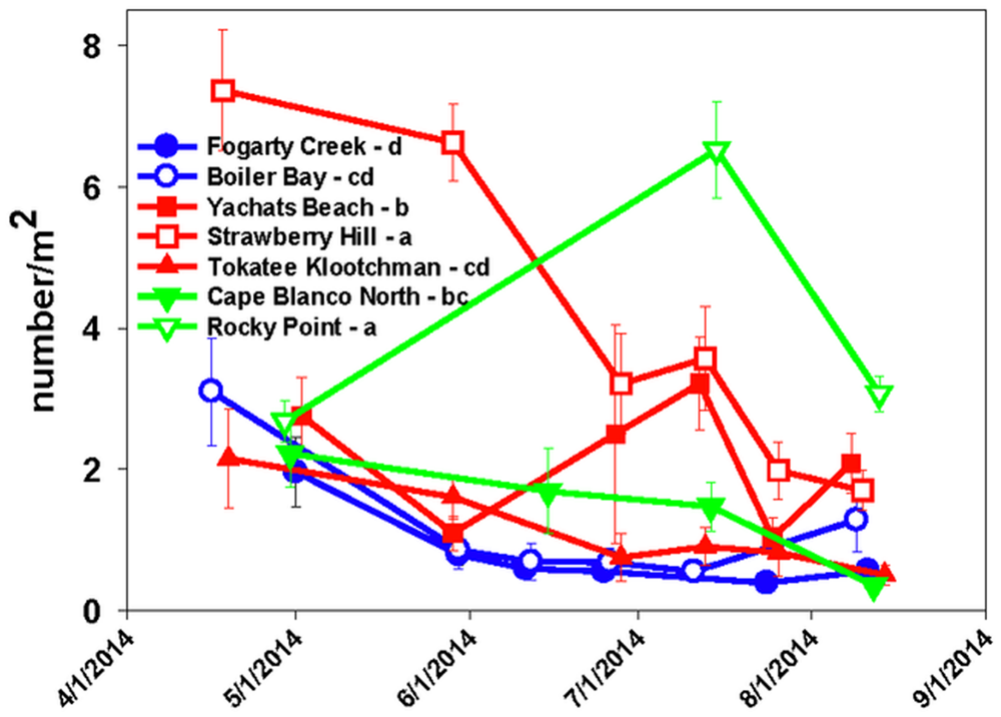
Decline in adult and juvenile *P*. *ochraceus* density, spring and summer 2014, at six sites. Differences in overall average densities (ln-transformed data in a 2-way ANOVA testing site and month without interaction term) are shown by lower case letters in legend, where sites sharing the same letter were not different at p > 0.05.

In summer 2014, *P*. *ochraceus* populations declined sharply except at YB and RP (Fig 5, Table 2). Increases in July at YB, SH and RP were likely driven by sand incursions, which typically occur at these sites from July to early fall (e.g., [36,37]). Sand can cover much of the lower intertidal rock surface, forcing *P*. *ochraceus* to move higher on the shore (B. Menge, pers. obs.). This upward movement tends to increase *P*. *ochraceus* transect numbers, artificially increasing densities. Water temperature can also cause variation in *P*. *ochraceus* density [31]. Cold upwelled water tends to inhibit activity and *P*. *ochraceus* tend to move downward whereas during warmer water events after upwelling cessation, sea stars tend to move upward towards mussel beds.

**Table 2:** Log-linear regressions of change in *P*. *ochraceus* density from April through August 2014^a^.

Site	Intercept	Slope	Quadratic term	SSE	MSE	F	p	R^2^
FC	266.95	-7.646e-8	1.71e-14(date-3.49e+9)	2.150	0.0538	15.44	**<0.0001**	0.407
BB	180.65	-5.17e-8	2.372e-14(date-3.49e+9)	4.0059	0.1145	14.10	**<0.0001**	0.415
YB	196.99	+5.676e-8	na	7.2389	0.2496	1.99	0.17	0.032
SH	456.74	-1.306e-7	na	2.9820	0.0904	66.25	**<0.0001**	0.657
TK	239.37	-6.846e-8	na	4.2908	0.1300	13.04	**0.001**	0.261
CBN	373.797	-1.07e-7	na	2.7178	0.1812	4.66	**0.047**	0.186
RP	145.28	-4.105e-8	-3.8e-15(date-3.49e+9)	0.4396	0.0366	20.03	**0.0002**	0.731

^a^Data were ln density (number/m^2^) and date.

Model p-values are in bold face.

na = not applicable.

**Fig 6:**
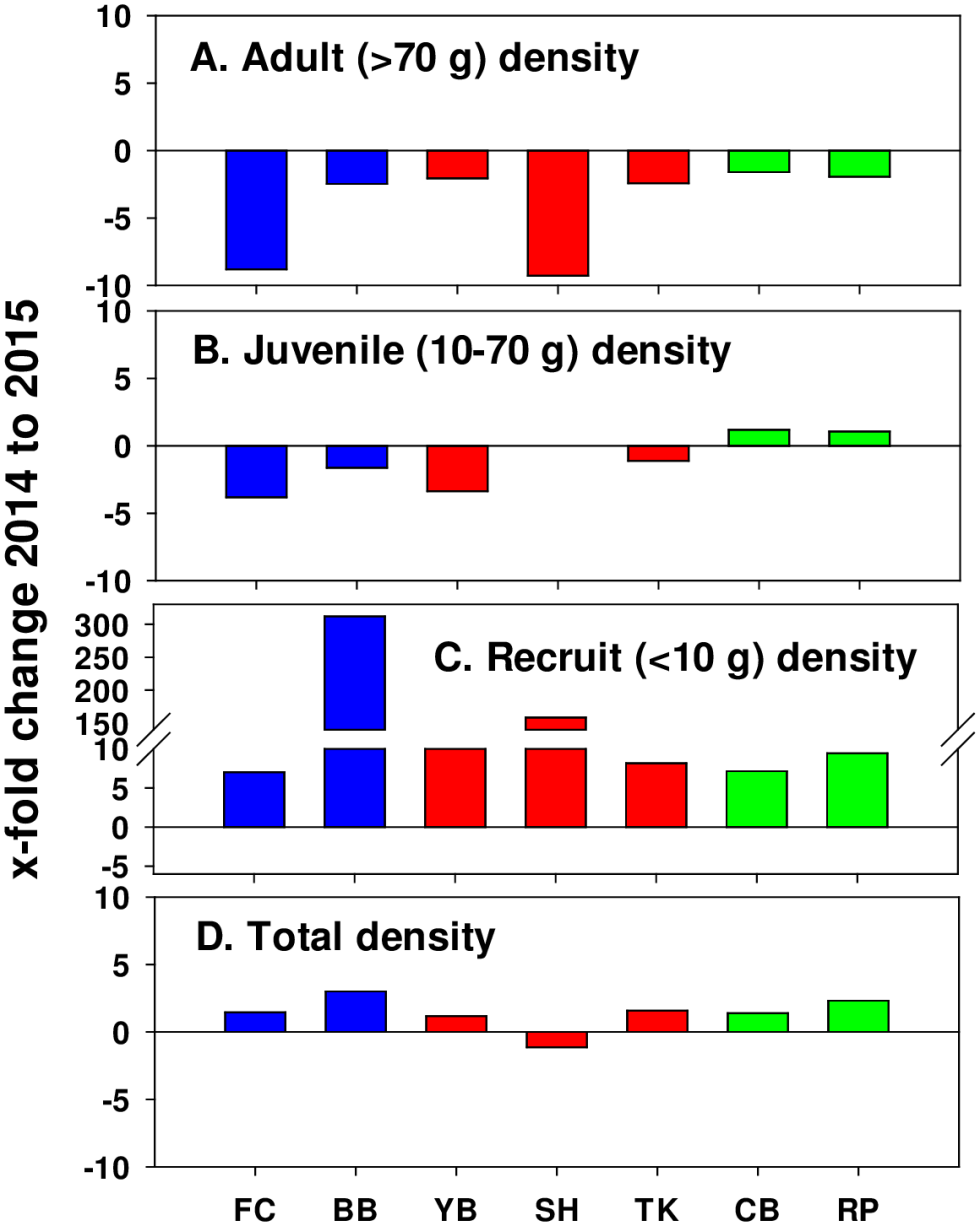
Magnitude of change from summer 2014 to summer 2015 in density (number/m^2^) of *P*. *ochraceus* at seven sites. Sites are color coded by cape, with blue = Cape Foulweather, red = Cape Perpetua, and green = Cape Blanco. A. Change in adult density, B. Change in juvenile density, C. Change in recruit density, D. Change in total sea star density. Weight ranges for each life history stage are approximate, and based on Menge 1974, which found that animals up to ~70g were non-reproductive and animals from ~100 g and larger had ripe gonads (before spawning). See Fig 2 caption for site names.

*P*. *ochraceus* density changes from 2014 to 2015 were driven primarily by decreases in abundance of adults across sites, with crashes as large as -8x to -9x (FC and SH; Fig 6A). Juvenile declines were smaller, and at CBN and RP juvenile numbers increased slightly in 2015 compared to 2014 (Fig 6B). Overall, however, total *P*. *ochraceus* density actually increased slightly in 2015 at all sites (except SH) because of exceptionally high recruitment (Fig 6C and 6D; see below).

SDS data included actual (or in summer 2015, estimated) measurements of sea star wet mass as well as numbers, enabling estimation of changes in *P*. *ochraceus* biomass (Fig J in S1 Appendix). In contrast to the sharp drops in density (Fig 5), biomass declined slightly from spring to summer at most sites in 2014 (presumably because most remaining individuals were still adults and juveniles). However, the largest declines occurred between years (Fig J in S1 Appendix inset; nested 3-way ANOVA, season p = 0.48, year p < 0.0001). The magnitude of decline from 2014 to 2015, and the proportion of biomass remaining in 2015 varied among sites (3-way ANOVA, p < 0.0001), ranging from a decline of -2.6x with 39% remaining at YB to a decline of -15.8x with 6.3% remaining at RP.

### Recruitment

**Fig 7:**
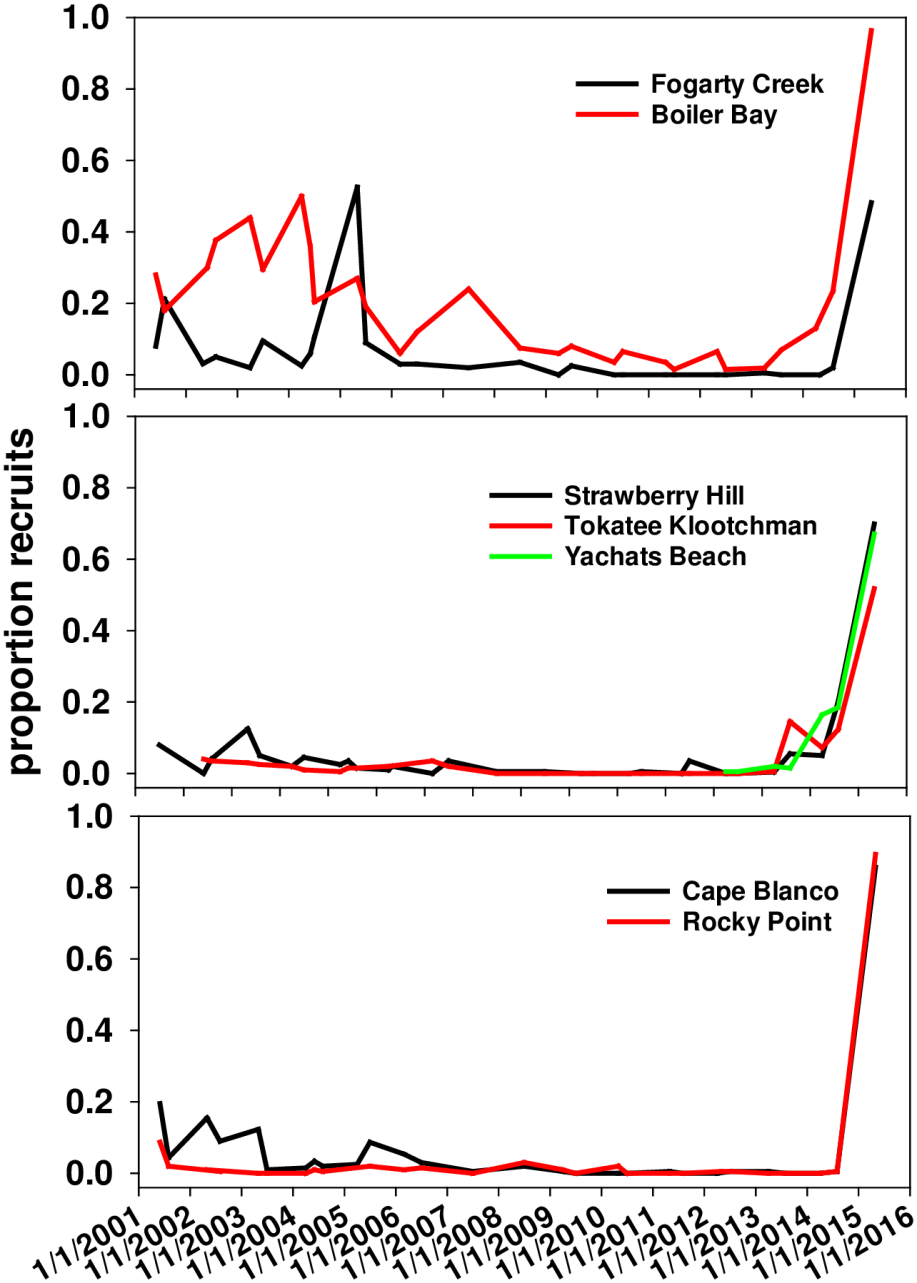
Average (+ 1SE) proportion of recruits of *P*. *ochraceus* over time at seven sites. Sample sizes were ~200 individuals per site per sample date. See Methods for details.

**Fig 8:**
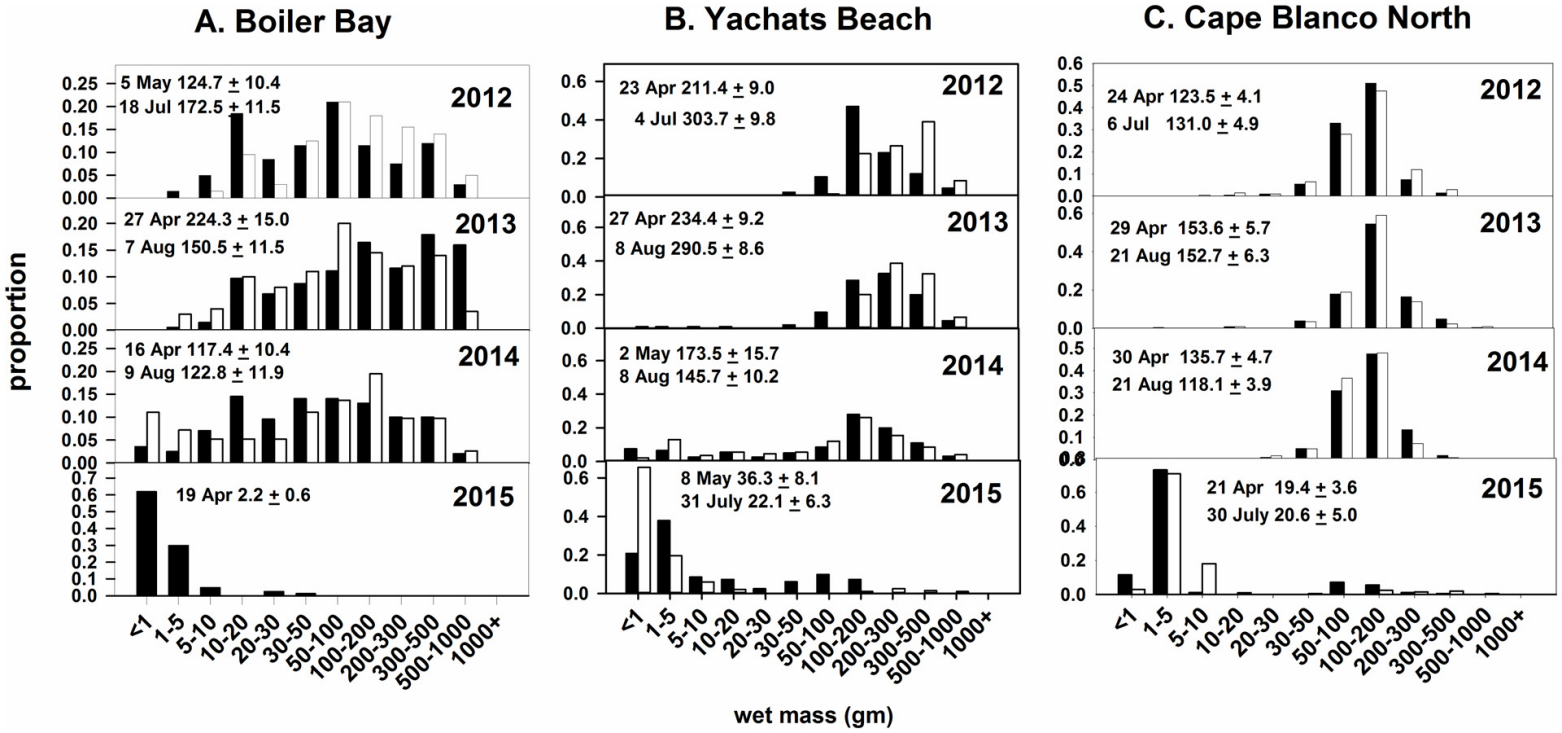
Size frequencies of *P*. *ochraceus* from 2012 to 2015 at three representative sites, one per cape. Samples were taken in spring (black bar) and summer (white bar) in each year except for Boiler Bay, where high wave action prevented collection of the summer 2015 sample. Sample size was ~200. Mean wet weight (± 1 SE) is shown for each sample date.

An unprecedented surge of *P*. *ochraceus* recruits occurred between fall 2014 and spring 2015 (Figs 7 and 8). Historically, the proportion of recruits in SDS was low at all sites except BB, especially in the years leading up to the wasting event (2009–2014; Fig 7). The 2014–15 declines in adults and juveniles and huge increases in recruits led to a dramatic shift in population size structure (Fig 8). Data for all sites but YB (begun in 2012) extend back to 2000–2001 at these and the other four sites, and show similar patterns (B. Menge, unpubl. data). Of the seven populations, only BB showed relatively regular annual recruitment (Fig 7) and relatively high juvenile frequencies (individuals 10 to ~ 100g; Fig 8A) before 2015. All other populations were similar to YB and CBN (Fig 8B and 8C), consisting mostly of adults (~100g or greater; [24,38]). SSWD-caused mortality and subsequent high recruitment led to a dramatic shift in size structure of *P*. *ochraceus* populations in favor of small, non-reproductive sea stars (Fig 8).

### Settlement

**Fig 9:**
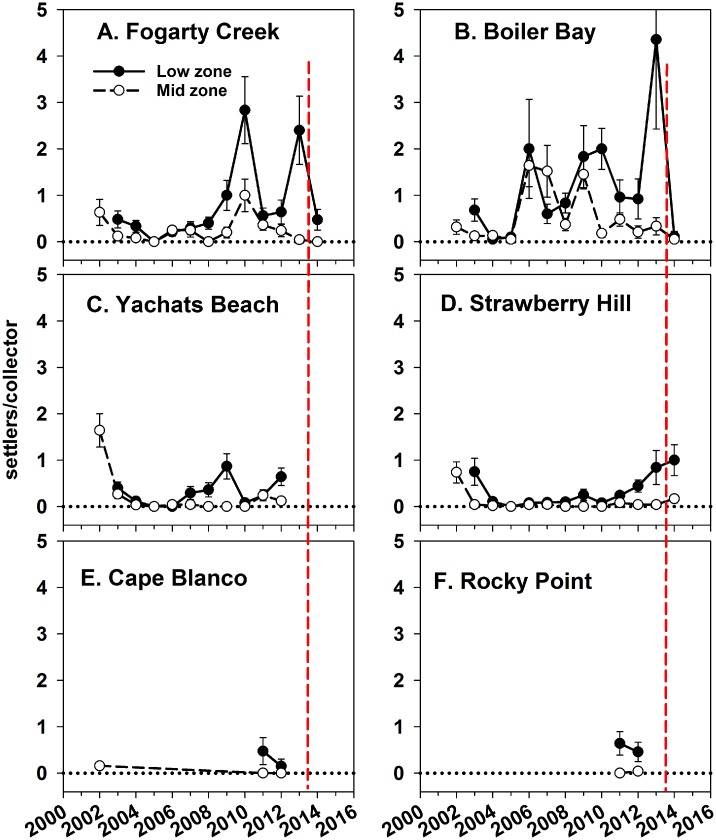
Annual average (± 1SE) number of *P*. *ochraceus* settlers at four sites. Vertical dashed line indicates the onset of SSWD.

In contrast to *P*. *ochraceus* recruitment, settlement patterns (i.e., individuals about 1–2 mm in diameter) were normal to low in 2014, at least at the three sites for which we have data (Fig 9; FC, BB and SH), and varied with site nested within cape, zone, and month (Table 3). Settlement in 2014 was lower than the previous four years at all sites combined (2010–13; linear contrasts, p < 0.0001), indicating that the heavy 2015 recruitment did not result from unusually high settlement in fall 2014. These observations indicate that recruits, as defined here, were ~6 to 9 months old (from the settlement stage; ~11–12 months old from the gamete stage).

**Table 3:** Test of effects of cape, zone, year and month on *P*. *ochraceus* settlement^a^.

Source	DF	Sum of Squares	F ratio	p
Cape	2	7.885150	54.7108	**<0.0001**
Zone	1	3.630847	50.3850	**<0.0001**
Year	12	15.644920	18.0920	**<0.0001**
Month[Year]	55	18.819677	4.7484	**<0.0001**
Zone x cape	2	0.638363	4.4293	**0.0120**
Site[cape]	3	1.378972	6.3786	**0.0003**
Error	2638	190.09964		**<0.0001**

^a^Adjusted R^2^ = 0.177.

DF = degrees of freedom.

### Predation Rate Experiments

**Fig 10:**
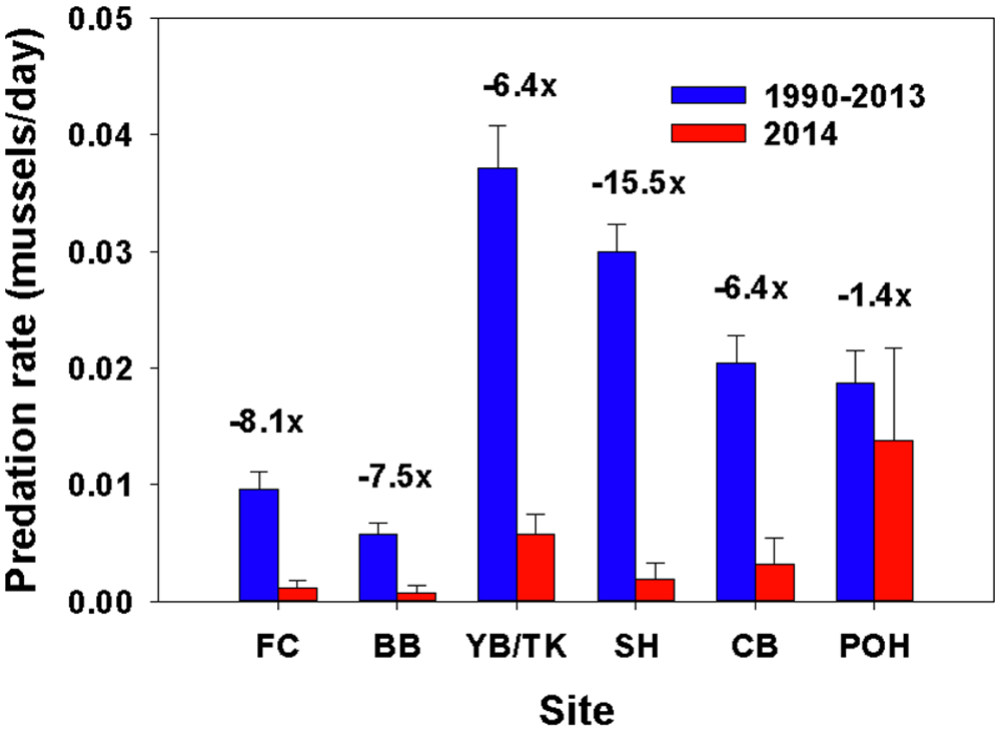
Comparison of predation rate on mussels, *Mytilus californianus*, in summer 2014 compared to rates averaged across 1990–2013 at six sites. Numbers above each pair of bars show the magnitude of decrease in predation rate.

SSWD sea star decline led to immediate effects on predation rate, with exceptionally low rates recorded in 2014 compared to all prior years in which the experiment was conducted, but with variation among sites (Fig 10; Table 4). Prior to 2014, predation rate was highest at CP sites, moderate at CB sites, and lowest at CF sites (linear contrasts among capes, p < 0.0001 in all comparisons). In 2014, however, predation rates did not differ among capes or sites (p = 0.28 or higher). Although summer 2014 numbers of *P*. *ochraceus* were still relatively high at some sites, particularly YB and SH (Fig 5), predation rate was severely depressed all summer at all sites except POH (Fig 10). Note that POH had the slowest rate of increase in wasting frequency of all sites (Fig 2C).

**Table 4:** Nested 2-way analysis of variance testing the effects of cape, site nested within cape, and era (prewasting vs wasting) on predation rates on transplanted mussels^a^.

Source	DF	Sum of Squares	Mean Squares	F Ratio	Prob > F
Cape	2	0.00491257	0.00245628	10.4622	**<0.0001**
Site[Cape]	4	0.00267417	0.00066854	2.8476	**0.0242**
Era	1	0.00598470	0.00598470	25.4911	**<0.0001**
Era x Cape	2	0.00208356	0.00104178	4.4373	**0.0126**
Error	313	0.07348495	0.000235	19.8290	**<0.0001**

^a^R^2^ = 0.345.

### Temperature

**Fig 11:**
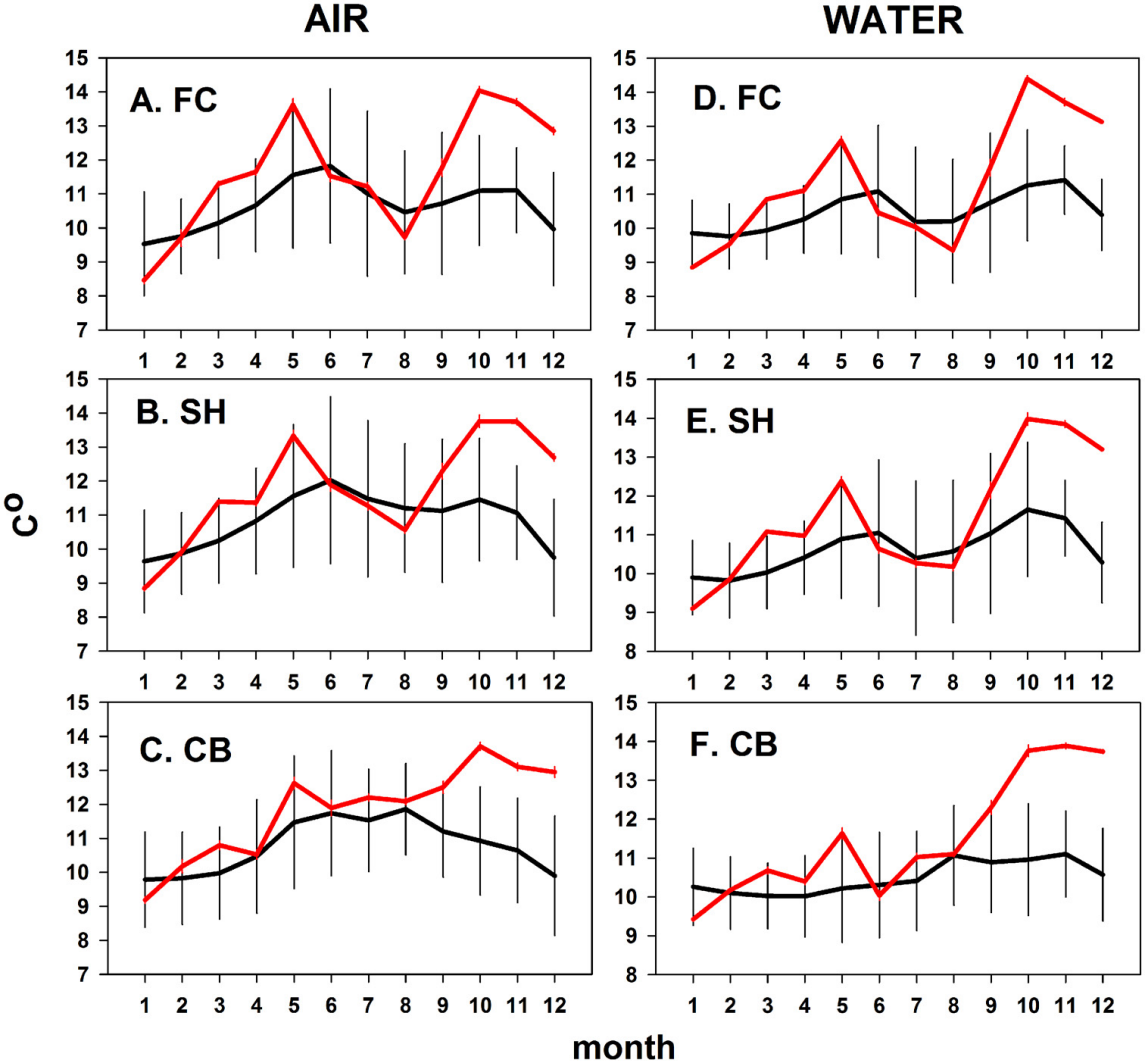
Air and water temperatures by month at three sites. Fogarty Creek and Cape Blanco North climatologies (black lines) were averaged from 1999 to 2014, and at Strawberry Hill from 1993 to 2014. The red line shows 2014 data. Climatologies are monthly means ± 1 SD and 2014 data are monthly means ± 1 SE.

Warm water and air temperatures have been linked to both previous wasting outbreaks (e.g., [20,21,39]), and to the current outbreak in Washington and southern California [3]. Air and water temperature climatologies (data ranges back to 1999 at FC and CBN, to 1993 at SH) in the Oregon intertidal varied among months and years (e.g., Fig 11). Annual mean air temperatures ranged from about 9.5 to 12°C while seawater temperature ranged from about 10 to 11.5°C. Overall, temperatures are higher in spring and early summer, decline in July and August when upwelling is strongest, and increase again in fall as upwelling ceases (Fig 11). As the standard deviations in Fig 11 show, however, cooler and warmer temperatures can occur most months.

Although temperatures spiked in May 2014, mean temperatures were not anomalous (i.e., different from past values as indicated by the 1 SD climatology window). At FC and SH, temperature declined in June, July and August, typical for the upwelling season. Exceptionally high temperatures were reached in October-December (Fig 11, red lines), but wasting % remained at about the same levels (Fig 2) even as sea star abundances were declining (Fig 5). Patterns at CBN were similar, with 2014 temperatures remaining in the 1 SD climatology window. Thus, in Oregon, although SSWD began after warm spring temperatures, its frequency increased during cold temperatures and was high during both cold and warm water and air temperatures.

Although data in Fig 11 are not consistent with a warm temperature effect on wasting, we examined temperature data in more detail to determine if the initially higher SSWD in the north (see **Wasting Surveys**) was related to temperature. Because relatively high temperatures were recorded in May and it was possible that this anomaly might have triggered the outbreak, we focused on April-June 2014 temperatures (Fig 11).

At the cape scale, air and water temperatures were partly consistent with a decline in temperature from north to south (Fig K in S1 Appendix, Table 5). In April, air temperatures did not differ among the three capes, but water temperatures were cooler at the southernmost CB (Table 5, linear contrasts). In May, water (and, more weakly, air) temperature declined from Cape Meares (north) to Cape Mendocino (south). With the onset of upwelling (indicated by summer drops in water temperature; Fig 11), June air and water temperatures among the three capes of focus were more idiosyncratic, with the highest temperatures at the central CP (Fig K in S1 Appendix, Table 5, air Cape * Month interaction, linear contrasts). Similar, but weaker, trends were seen at the site scale (data not shown).

**Table 5:** Two-way analyses of variance testing effects of cape and month on intertidal air and water temperatures at Capes Foulweather, Perpetua, and Blanco for April-June 2014.

Medium	Source	df	Sum of Squares	F	p	Adj R^2^
Air	Cape	2	9.8825	2.12	0.12	0.2204
	Month	2	535.556	114.9	**<0.0001**	
	Cape x Month	4	25.7915	2.77	**0.026**	
	Error	898	2092.0602			
Water	Cape	2	139.614	55.10	**<0.0001**	0.3717
	Month	2	512.0146	202.1	**<0.0001**	
	Cape x Month	4	11.98975	2.37	0.051	
	Error	901	1141.513			

We also examined if extreme daily temperatures in April 2014 might be consistent with (as a “trigger”) a north-south gradient in wasting. Maximum daily air temperatures recorded at CF (north) were 14.12°C (FC) and 13.15°C (BB); at CP (central) were 12.07°C (YB), 14.25°C (SH) and 15.46°C (TK); and at CB (south) were 14.03°C (CBN), 12.25°C (POH), and 18.3°C (RP). Maximum water temperatures ranged from 11.66°C to 13.91°C and were similarly intermingled among capes. Thus, while temperatures were approximately consistent with a north-south gradient at the cape scale, no trend was evident for maximum daily temperatures.

Since mean temperatures may not capture variability in temperature or exposure to extremes, we also examined the relationship between these factors in climatologies vs. 2014. These comparisons were consistent with results for monthly mean temperatures; neither the patterns of variability of air and water temperature nor the maximum-minimum envelopes showed unusual patterns in 2014 compared to long-term values (Figs L and M in S1 Appendix). The only exception was that the maximum for May 2014 was slightly higher than the climatology maximum for May; all other 2014 summer maxima were well within the climatology envelopes.

### pH

The coast-wide occurrence of SSWD suggests that if some environmental factor triggered the disease outbreak, it was also a coast-wide event. A steadily increasing factor along the coast (and in the ocean at large) is ocean acidification (e.g., [40–46]). In 2014, pH and temperature data were collected every 10 minutes intertidal and subtidal sensors at SH (Fig N in S1 Appendix). These data show that at 4 m depth offshore, sea water temperature slowly increased from about 10 to about 14°C into mid-May, 2014, then dropped sharply around the 21^st^ of May. pH changes were similar, remaining around 8.1, then dropping rapidly to about 7.8 in mid-May. The pH then varied, reaching about 7.6 in early June while temperatures changed concurrently but with less variability. Intertidal temperatures and pH varied similarly, with drops in both matching those observed on the mooring sensors.

**Fig 12:**
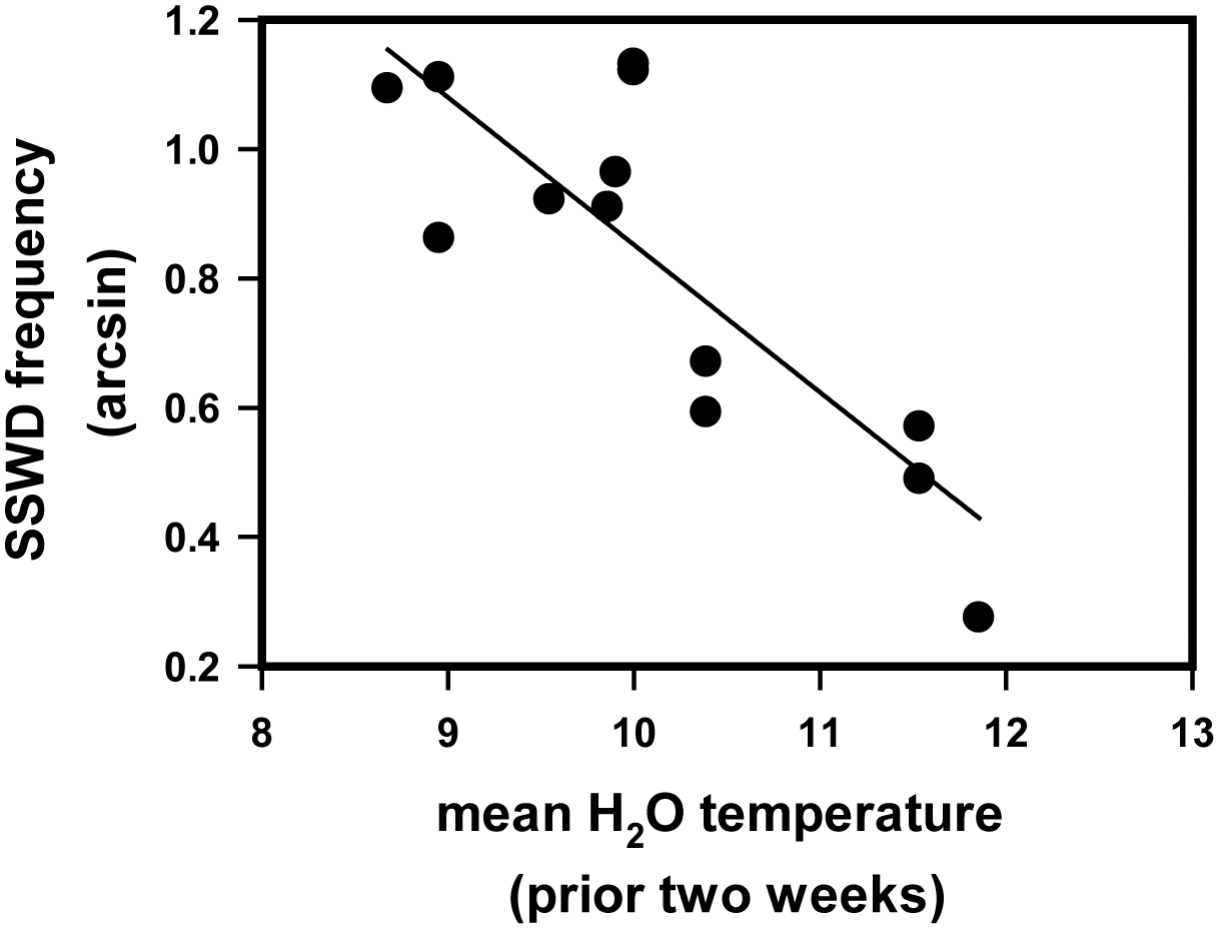
Log-linear regression between water temperature averaged over the previous two weeks of the sample date and the frequency (arcsin-transformed) of SSWD on the sample date.

Because datasets were most complete at SH, we quantified the relationship between pH, temperature and the proportion of the population at this site with wasting disease using stepwise multiple regression. The best fit model (AICc) included only temperature averaged over the previous two weeks (Fig 12, wasting frequency = 3.1323–0.228[average water T previous two weeks]; p = 0.0004, R^2^ = 0.668, AICCc = -4.197). Wasting frequency was inversely related to temperature but unrelated to pH.

## Discussion

Our results provide critical insight into one of the largest marine disease events ever recorded. From spring 2014 to summer 2015 in Oregon, SSWD caused drastic population declines between 63 and 84% (adult density) and 80 to 99% (biomass) in *P*. *ochraceus*, comparable to those seen elsewhere along the US west coast (3). Further, SSWD-caused declines in predation rates by sea stars on *Mytilus californianus*, together with insights from classic experimental studies [8,10], suggests that community structure may change dramatically throughout the region. Our detailed and extensive chronology of the disease outbreak was only possible because of the ongoing long-term research at these sites, and exemplifies the value of funding long term monitoring. Our ability to document the epidemic as it occurred was facilitated by the earlier reports of wasting from Washington, British Columbia and southern and central California (e.g., http://www.eeb.ucsc.edu/pacificrockyintertidal/data-products/sea-star-wasting/) and the relatively slow progression and persistence of the disease. Though *P*. *ochraceus* populations were severely affected, a surge in recruitment of young sea stars occurred a few months after the outbreak. It does not appear that the disease outbreak triggered high settlement, but it potentially indirectly facilitated high recruit survival. While the recruitment event may facilitate recovery, the disease is ongoing and the loss of reproductive adults suggests that the supply of propagules will remain low for years. Hence, the pace of recovery of *P*. *ochraceus* populations likely hinges on the survival of these recruits.

### Variation in Wasting

We highlight several novel results. SSWD varied with age/size, and subhabitat. For example, animals in tidepools were more susceptible than those out of tidepools. Hypothetically, greater susceptibility in tidepools could result from the longer exposure to water-borne pathogens. However, individuals out of tidepools are likely to become warmer and more stressed during low tide [45–47], which could decrease immune function [1]. Thus, the causal relationship between susceptibility and microhabitat is unclear.

Counterintuitively, during much of 2014 summer, susceptibility was lower in juveniles compared to adults (Fig 3, adults = 55.3 ± 2.6% vs. juveniles 3.9 ± 0.9%). The mechanistic basis for this apparent resistance is unclear and under investigation [1,11]. Possibilities could include: (1) higher frequencies of resistant genotypes or phenotypes among juveniles and recruits, (2) physiological differences between adults and small sea stars that make adults more susceptible to SSWD, (3) higher surface areas and larger volumes of water within the water vascular system in adults to “intercept” or retain water-borne pathogens, respectively, and (4) small size may make recruits harder to detect because they may die “faster.”

Finally, wasting varied in timing, first occurring most intensely northward and spreading southward. The rate of increase varied among capes and sites, but disease frequency persisted at a high rate (~60–80%) into the late fall. SSWD frequency declined over winter and persisted but at low levels in spring and summer 2015 except at two sites, where a fresh outbreak began in late summer. The mechanistic basis of this trend is unclear.

### Ecological Consequences

Unlike some prior wasting events (e.g., [19–21,39]), this event was coast wide, ranging from Alaska to Baja California. It thus ranks among the most extensive disease events ever recorded for a marine species [2,3]. Its consequences may be among the largest coastal-scale biological disruptions to a large marine ecosystem, joining sea star die-offs from 1982–83 and 1997–98 El Niño events, the die-off of sea stars (*Heliaster kubinjii*) in the Gulf of California in the 1970’s, of sea urchins (*Diadema antillarum*) in the Caribbean in the 1980s, and the historic human-caused extirpation of sea otters (*Enhydra lutris*) across much of their Pacific coastal range (e.g., [18,48–51]).

A drastic reduction in predation rate was an immediate consequence of SSWD (Fig 10). A hallmark of prior experiments ranging back to 1990 was site-specific consistency of predation rate up to ~2009 (e.g., [52]). Although predation rates differed among sites, annual rates at each site tended to be similar. In summer 2014, however, even though some sites retained large numbers of asymptomatic animals into mid-summer (Fig 5, SH, YB, and RP), predation rate dropped sharply. This change was not likely to have been due to cold water temperatures, which occurred during the height of SSWD. Although prior research showed that cold water temperatures (e.g., those occurring during summer upwelling) can inhibit feeding, *P*. *ochraceus* can acclimate and if cold water events persist, feeding rates will increase ([31,32]). Further, unusually low predation rates persisted into fall 2014 when waters had warmed after the cessation of upwelling (B. Menge unpubl. data). We conclude that the steep drop in predation rate was driven by SSWD, with behavioral effects possibly occurring before external symptoms of the disease were apparent.

Based on prior research [8,10], the longer-term ecological consequences of this SSWD event could include wholesale elimination of many low zone species and a complete change in the zonation patterns of rocky intertidal communities along the west coast of North America. In other words, *P*. *ochraceus* loss may shift the low zone to an alternate community state dominated by mussels, which could potentially persist for years to decades [53]. If mussel abundance change does track the “alternate state” hypothesis, it would result in losses or large reductions of many species of macrophytes, anemones, limpets, chitons, sea urchins and other organisms from the low intertidal zone. For many of these species presence in the low intertidal zone represents upward extensions of their subtidal ranges (e.g., [54,55]), so unless mussels also invade the subtidal (which is a possibility), they are likely to persist subtidally. Other species, however, are restricted to the intertidal (e.g., *Saccharina sessilus*, *Katharina tunicata*) and may not persist in a mussel-dominated zone. *M*. *californianus* shells typically provide substratum for many other organisms (“epibionts”), including barnacles, *M*. *trossulus*, limpets, herbivorous gastropods, and some macroalgal species (B. Menge unpubl. data). Further, the “byssal forest” under the mussel bed provides shelter for dozens of species [56,57], so many taxa may actually benefit from the expected expansion of the mussel bed into the low intertidal zone. Overall, although it is likely that community structure will change considerably, how spatially “universal” (i.e., widespread along the coast) this effect will be is unclear.

An alternative to changes occurring uniformly along the coast is a “mosaic” response. That is, the low intertidal zone will become dominated by *M*. *californianus* at some sites but not at others. For example, mussels might dominate at wave-exposed areas, but not at wave- sheltered ones. Many complex interactions occur within *P*. *ochraceus*-dominated low intertidal communities, and these could also influence the rates or direction of change. Thus, mussels may lose dominance at wave-exposed areas where alternative predators become more influential than at present in affecting intertidal community structure. Alternative predators in this system include whelks (*Nucella ostrina*, *N*. *canaliculata*), the small sea star *Leptasterias* spp., crabs (e.g., *Cancer productus*), and sea gulls (*Larus* spp.)[33,58–66]. Facilitation and competition for space, food or light may also occur among various components of the low zone intertidal causing unexpected community responses [23,37,67–69]. Finally, mussels will only become dominant if they recruit to a location, and recruitment of mussels is highly variable in space [70]. Because wave exposure, compensatory predators, other species interactions, and mussel recruitment may all influence diversity loss and shifts in abundance and species composition, we expect a mosaic response rather than a wholesale shift to an alternate state along the California Current Large Marine Ecosystem (CCLME).

The near coast-wide appearance of unprecedented numbers of recruits in 2015 suggests that *P*. *ochraceus* population recovery may be rapid, but additional considerations could lead to slow recovery. As of summer 2015, we observed that the cohorts of recruits appeared to be growing rapidly (perhaps due to a surfeit of small barnacles in the low intertidal zone), and were achieving “juvenile” status. At this rate, a new cohort of (presumably) reproducing adults may mature within several years on many shores. If, on the other hand, SSWD continues to kill a high proportion of individuals in the coming years, it is possible that many of the current cohort of recruits will also die off. With the low abundance of adults following SSWD and the slow maturation time in this species, the number of propagules released will likely be low for several years potentially delaying population recovery.

What are the likely implications over the longer-term, or larger-scale? If sea star recovery is slow, taking many years, then it seems likely that mussel invasion of the low zone will be widespread. Precedents for quick recovery, slow recovery, and no recovery exist for sea star mass mortality events. In the New England subtidal, Witman et al. [71] showed that after a huge pulse of mussel recruitment, abundance of predators (mostly the sea star *Asterias vulgaris* but also crabs) increased and in < 2 years, reduced mussel abundance back to normal, near 0% cover. In southern California, *P*. *ochraceus* populations in the Channel Islands recovered from severe depletion after the ENSO event of 1997–98 within about 7 years [21]. In this case, however, nearby populations of *P*. *ochraceus* were unaffected, thus providing a potential source of new recruits for Channel Island populations. The “no recovery” example is the virtual extinction of *Heliaster kubinjii* in the Gulf of California due to SSWD mentioned earlier [18]. It is unclear which of these examples might apply to the present case. If *P*. *ochraceus* adults become scarce along most of their range, then juvenile recolonization may be limited by a lack of reproducing adults due to low numbers of gametes released and/or low odds of gamete fertilization. Since planktonic larval mortality is typically very high, e.g., 99% or more (e.g., [24]), recovery of *P*. *ochraceus* populations may be hindered by a larval abundance bottleneck.

### Symptomatology

Twisting or contorting arms and lesions were generally the most common symptom (Fig I in S1 Appendix; see also [1–3]), and our observations suggest that arm twisting preceded lesions and other symptoms (Fig 4). Deflation tended to follow arm twisting, but never was as common as lesions. It is not clear if deflation was an inevitable part of the progression of SSWD, but its mostly low frequencies suggest that only some afflicted animals show this symptom. Based on its low frequencies, losing arms may also be a symptom that only some portion of the afflicted animals displayed. Indeed, we observed many disintegrating sea stars that still possessed 5 arms.

For animals ultimately dying of SSWD, losing grip and disintegration likely are inevitable symptoms. Both declined in frequency toward the end of the summer and into the fall (Fig 4M–4R), which is consistent with the major mortality impact of SSWD occurring during mid-summer. These symptoms can progress very fast; we observed one adult animal as “losing grip” early in a tide, and “disintegrating” only four hours later (Fig A in S1 Appendix). Hence, it seems likely that relatively few observations of disintegration is because this stage is brief, and that wave action during submergence likely washes remains of diseased individuals away.

### Changes in Abundance

SSWD caused severe declines in adult and juvenile *P*. *ochraceus* populations at nearly all sites. Although density time series going back to 2001 show that density has fluctuated through time at all sites (B. Menge, unpubl. data), changes as persistent and negative as those observed during 2014 have not been seen in > 30 years (since 1982–83). Sites where declines were less severe (YB and RP) still had high disease frequencies, and as suggested earlier we believe the apparent lack of declines were driven by factors driving sea stars upward including warm temperatures (e.g., [31]), and sand incursions. We strongly doubt that alternate sources of mortality caused abundance changes, because many years of research suggest that *P*. *ochraceus* mortality is normally very low and is due to the occasional animal being detached from rock and tumbling in the surf or being captured by sea gulls. We have observed no other predators on *P*. *ochraceus* (sea otters do not occur along the Oregon coast), and have never, in literally 10s of thousands of observations, seen cannibalism among or between adults, juveniles, or recruits, either in the field or laboratory. Indeed, *P*. *ochraceus* commonly tend to aggregate in large masses, especially around mussel patches. In these masses and in channels and crevices, adults are often atop juveniles and recruits, with no sign of impending predation (e.g., no everted stomach, no damage). We conclude that the sharp drops in adult and juvenile density and population biomass were driven entirely by SSWD.

### Population Replenishment

What was the cause of the unprecedentedly high recruitment in spring 2015? The most likely mechanism is that reduced *P*. *ochraceus* abundance led to increases in successful mussel and barnacle recruitment. In Oregon, dense barnacle and mussel recruitment typically occurs each year from July through November [23,52], coincident with the early benthic life history stage of *P*. *ochraceus*. Thus, we hypothesize that in previous years, competition with adults for food suppressed survival of settled *P*. *ochraceus*, accounting for the usual low sea star recruitment from at least 2001 to 2014 (Fig 7). Of course, the orders of magnitude difference in size of the recruits and adults means that adults are likely to prefer larger prey [72]. However, adults are often observed with small barnacles and mussels in their everted stomachs, especially when larger prey are scarce (fall and winter), so they may deplete prey when recruits are most vulnerable to starvation. Such a possibility has a precedent. Along the coast of Maine, prey-driven pulses of mussel recruitment were proposed as the explanation for high recruitment of sea stars in rocky subtidal habitats [71]. More research is needed to clarify the degree of competition between adult and recruit *P*. *ochraceus* for small barnacle and mussel prey.

An alternative possibility for high survival might be relaxation of predation. This explanation is unlikely given the lack of observed predation or cannibalism on *P*. *ochraceus* juveniles. Possible physical causes of the 2015 recruitment surge also seem implausible. Although ocean temperatures along the US west coast have increased [73,74] and pH is likely decreasing (e.g., F. Chan et al. unpub. data), these changes have been gradual, not sudden like the recruitment pulse. A final possibility is that the recruit pulse was a chance event. Historically, some echinoderms have been shown to have highly pulsed recruitment [75,76] with no obvious cause. However, the widespread dense *P*. *ochraceus* recruitment along the entire coast suggests random chance is unlikely.

### Possible Factors Underlying Disease

The proximate factor causing wasting likely includes infection by a densovirus (SSaDV; [2]). SSaDV occurs in high abundance in infected animals, and inoculation with a viral-sized fraction experimentally and exposure to it in sea water (through co-habitation with infected individuals) both generated wasting in sea stars in lab experiments. However, SSaDV was evidently present in all sea stars tested, even asymptomatic individuals, and occurred in the surrounding water column [2]. It was also found in museum specimens preserved as early as 1942, so has been present in these systems for decades. Thus, whether SSaDV suddenly increased in abundance, overwhelming the immunological systems of sea stars, or increased in virulence is unclear. Hewson and colleagues are pursuing these questions and continuing the search for additional or complementary pathogens in SSWD (I. Hewson, personal communication).

High densities of sea star populations has been suggested as a possible factor underlying the SSWD outbreak off Vancouver, BC [1]. In Oregon, however, SSWD frequencies were high at all sites during summer, despite the widely different *P*. *ochraceus* densities (Figs 2 and 5).

What are likely environmental changes that could have contributed to the recent outbreak of wasting disease in Oregon? As our temperature and pH analyses suggest (Fig 12, Figs K-N in S1 Appendix), evidence for climate as a direct trigger of wasting is not supported by our data. Instead, biological factors, for example changes in the abundance or virulence of SSaDV or changes in host susceptibility may be either interacting indirectly with environmental triggers in the outbreak or independently of any environmental change. Environmental changes could influence disease transmission or host susceptibility if sea stars were experiencing increased stress and lowered immune response. Although unusually warm temperatures have been cited as a co-varying factor associated with SSWD outbreaks in Washington (3), British Columbia [39], and southern California [20,21], Oregon temperatures were not unusually high in 2014, and were cooler than most previous years during the height of the outbreak (Fig 11). In fact, wasting frequency was most strongly related to cooler, not warmer temperatures (Fig 12). Water temperatures in central California (Monterey Bay area) were also cooler than usual in fall 2013 when the outbreak occurred, not warmer (P. Raimondi, pers. comm.). We further note that none of the temperatures we recorded were close to those found to be stressful in lab and field studies [45–47,77–80]. Although the May 2014 air and water temperatures were higher than average, in previous years similar relatively high temperatures have been reached in most summer months (e.g., Fig 11, [31,81]) with no evidence of wasting. Finally, the truly exceptional high temperatures in autumn 2014 did not appear to lead to a change in SSWD, and in fact, SSWD frequency declined over the subsequent months.

Ocean acidification is also intensifying ([41], F. Chan et al. unpublished data), but at SH, pH and temperature co-varied (linear regression; mean pH previous week = 6.19 + 0.18(mean T previous week); p <0.0001, adj R^2^ = 0.797, n = 13). Although our analysis suggested only temperature was related to SSWD (and inversely, not positively), it seems likely that the ultimate explanation of SSWD is multifactorial, involving an as yet unknown combination of environmental and biological factors.

### Comparison to Other Results

Eisenlord et al. [3]reported results of their research in Washington, and concluded that, in contrast to our analysis, warm temperatures were an important driver of SSWD in the sheltered waters of the San Juan Islands, with weaker effects in South Puget Sound. Consistent with our results, their analysis suggested large animals were more susceptible than small ones. At one site on the Washington outer coast (Starfish Point) SSWD prevalence ranged up to 62% but peaked in winter 2015 in contrast to late summer and early fall in the San Juan Islands. Data from this site, probably most comparable to our Oregon sites, were insufficient to analyze a relationship with temperature. Although their conclusion of an impact of warm temperatures is consistent with prior suggestions, the association of high SSWD with cool conditions in Oregon (and Eisenlord et al.’s observation of high frequency of SSWD at their open coast site in winter, when ocean and air temperatures were likely low) raises questions about the generality of the relationship between temperature and wasting. We can envision three explanations: (1) SSWD is unrelated to temperature, (2) multiple pathogens or pathogen genotypes with differing environmental sensitivities are involved and vary geographically (i.e., different forms occur in outer and inner coasts, latitudinally along the coast, etc.), or (3) some other factor, whether intrinsic or extrinsic, is a key factor. Much remains to be learned.

## Conclusions

SSWD clearly caused dramatic decreases in population density and biomass along the Oregon coast, with large and immediate effects on the predation rate of by *P*. *ochraceus* on mussels at all sites. Initially, adults in tidepools were most strongly affected. Populations were sharply less abundant by late summer 2014. As observed at many locations along the US west coast, a new cohort of *P*. *ochraceus* recruits were detected in spring 2015, dramatically altering the size structure of populations and underlying a recovery in numbers but not biomass. By spring 2015 had declined to moderate levels, ranging from ~2–15% at all sites but two (SH, TK), where new outbreaks appeared in late summer 2015. The mechanism enabling high recruitment could have been a high abundance of small prey (barnacle and mussel recruits), resulting from depletion of adult and juvenile populations. Given conflicting results on the role of temperature as a trigger of SSWD, it seems most likely that multiple factors interacted in complex ways to cause the outbreak.

## Supporting Information

S1 Appendix Fourteen supporting figures. Figures show photos of sea stars with wasting symptoms, the proportion of symptoms at 9 sites, the decline in biomass during the wasting outbreak, cape-scale mean air and water temperatures for April to June 2014, comparison between the air and water standard deviations by month in 2014 to the long-term climatology, comparison between maximum-minimum air and water temperatures in 2014 to the long-term climatology, and a comparison between water temperature and pH onshore and on an offshore mooring at Strawberry Hill.
(DOCX)
