# Correction: Sea Star Wasting Disease in the Keystone Predator *Pisaster ochraceus* in Oregon: Insights into Differential Population Impacts, Recovery, Predation Rate, and Temperature Effects from Long-Term Research

**DOI:** 10.1371/journal.pone.0157302

**Published:** 2016-06-03

**Authors:** Bruce A. Menge, Elizabeth B. Cerny-Chipman, Angela Johnson, Jenna Sullivan, Sarah Gravem, Francis Chan

Table 1 is incorrect. The authors reanalyzed the color and subhabitat data and found that wasting did not vary with color, as originally concluded. As a result, some of the related text was also incorrect. Please find the corrected table and text below.

**Table 1:** Comparison of percentages of total number of *P*. *ochraceus* that were “normal” or “wasting” (shown in paired columns) in all combinations of color and subhabitat by month, summer 2014. Boldface numbers show month pairs that were different and how differences changed through the summer, across months (tested with linear contrasts on months after two-way anova on difference between wasting and normal arcsine-transformed percentages by cape and month; cape was never significant).

Month (2014)	N	Normal Orange	Wasting Orange	Normal Purple	Wasting Purple	Normal Pool	Wasting Pool	Normal Out of Pool	Wasting Out of Pool
May	7	18.2 ± 1.0	23.9 ± 3.0	81.8 ± 1.0	76.1 ± 3.0	**27.9 ± 5.4**	**43.9 ± 7.8**	**72.1 ± 5.4**	**56.1 ± 7.8**
June	13	21.9 ± 2.0	21.4 ± 1.4	78.1 ± 2.0	78.6 ± 1.4	23.1 ± 4.4	31.2 ± 4.2	**76.9 ± 4.4**	**68.8 ± 4.2**
July	11	19.9 ± 2.1	23.6 ± 1.2	80.1 ± 2.0	76.4 ± 1.2	16.1 ± 1.8	21.8 ± 3.5	83.9 ± 1.8	78.2 ± 3.5
August	11	22.5 ± 2.5	22.6 ± 1.8	77.5 ± 2.5	77.4 ± 1.8	11.1 ± 2.0	10.5 ± 2.4	88.9 ± 2.0	89.5 ± 2.4
September	7	20.1 ± 1.9	20.6 ± 2.1	79.9 ± 1.9	79.4 ± 2.1	14.2 ± 3.8	9.0 ± 2.5	85.8 ± 3.8	91.0 ± 2.5

There is an error in the seventh sentence of the Abstract. It should read: SSWD was disproportionally higher in individuals in tidepools.

There are errors in the fourth paragraph of the Results. It should read: In the early summer, adult sea stars in different sub-habitats were differentially susceptible to SSWD (Table 1). Purple sea stars made up ~80% and orange individuals 20% of all populations, and sea stars tended to be found more often outside (~73%) than inside (~27%) tidepools. If sea stars across color and sub-habitat combinations were equally affected with wasting, the proportion of each color or sub-habitat among asymptomatic (“normal”) and symptomatic (“wasting”) should be similar. No differences were observed between normal and wasted color proportions, but early in the April-November field season, higher proportions of wasted animals occurred within than outside of tidepools (Table 1). By July, no differences related to subhabitat were detected. As suggested by the changing proportions in successive months in Table 1, these changes seem likely due to the declines in numbers of differentially susceptible sea stars.
