# Supplementary material for: Sea Star Wasting Disease in the Keystone Predator Pisaster ochraceus in Oregon: Insights into Differential Population Impacts, Recovery, Predation Rate, and Temperature Effects from Long-Term Research
Source: PLoS One. 2016 May 4;11(5):e0153994. doi: 10.1371/journal.pone.0153994 (PMC4856327; doi:10.1371/journal.pone.0153994)
Supplement: S1 Appendix — Figures show photos of sea stars with wasting symptoms, the proportion of symptoms at 9 sites, the decline in biomass during the wasting outbreak, cape-scale mean air and water temperatures for April to June 2014, comparison between the air and water standard deviations by month in 2014 to the long-term climatology, comparison between maximum-minimum air and water temperatures in 2014 to the long-term climatology, and a comparison between water temperature and pH onshore and on an offshore mooring at Strawberry Hill. (DOCX) [file pone.0153994.s001.docx]

**S1 Appendix. Fourteen supporting figures.** Figures show photos of sea stars with wasting symptoms, the proportion of symptoms at 9 sites, the decline in biomass during the wasting outbreak, cape-scale mean air and water temperatures for April to June 2014, comparison between the air and water standard deviations by month in 2014 to the long-term climatology, comparison between maximum-minimum air and water temperatures in 2014 to the long-term climatology, and a comparison between water temperature and pH onshore and on an offshore mooring at Strawberry Hill.


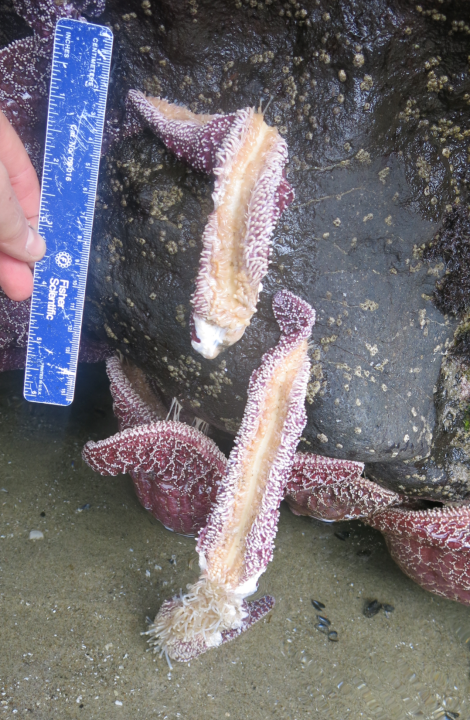

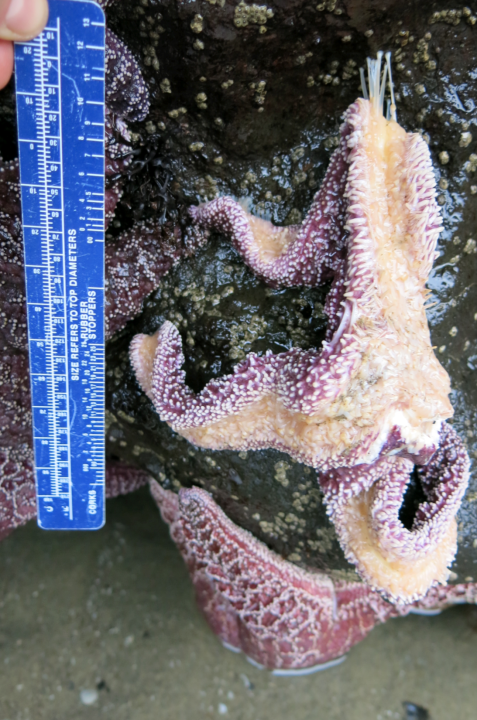


**10:20 AM**

**6:20 AM**

Fig A. Example of the rapidity with which wasting can occur. An animal losing its grip on the rock at 620 am had largely disintegrated four hours later. Observations made at Cape Blanco South (see Fig 1).


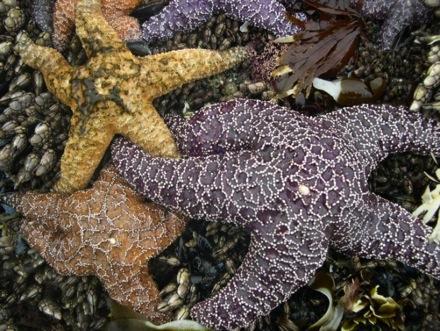


Fig B. Photo of *Pisaster ochraceus* with affliction ranking 0: Asymptomatic. The yellowish individual has diatoms attached to its spines, which is commonly observed and not a symptom of wasting.


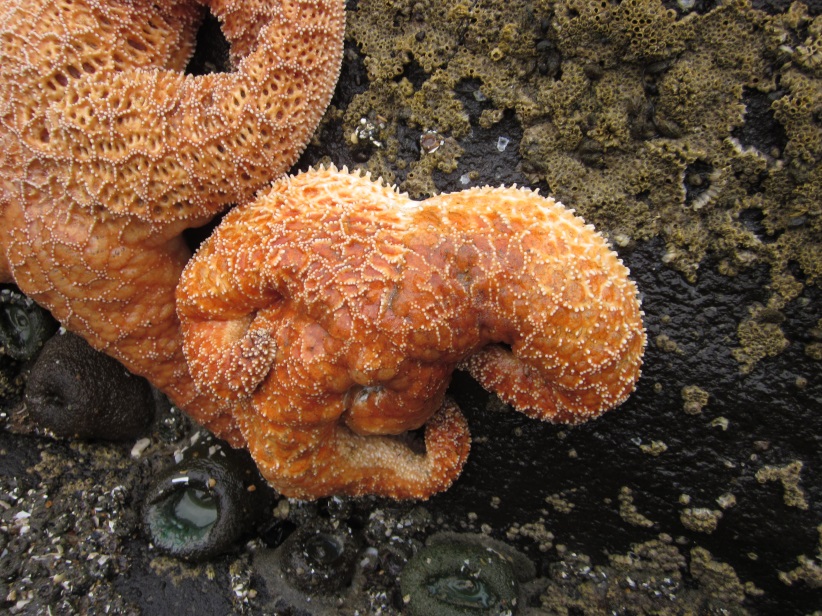

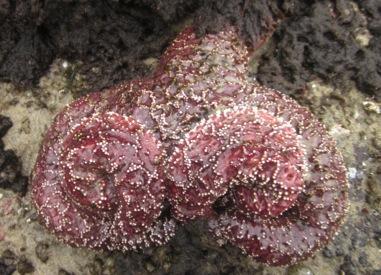


Fig C. Photos of *Pisaster ochraceus* with affliction ranking 1: Symptomatic. Animals with “twisting” arms. The orange and bright purple individuals in the left photo and the purple individual in the right photo show two examples of arm twisting, which is not seen in asymptomatic individuals.


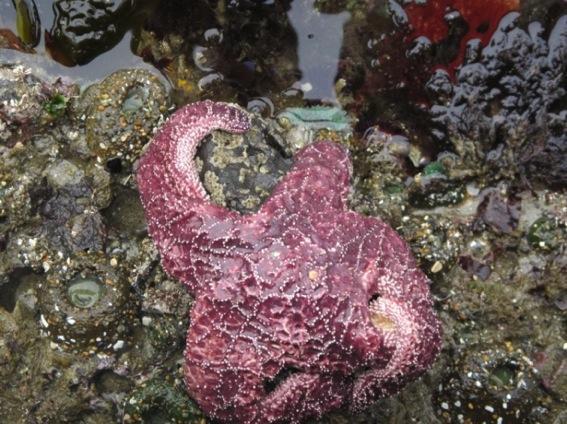


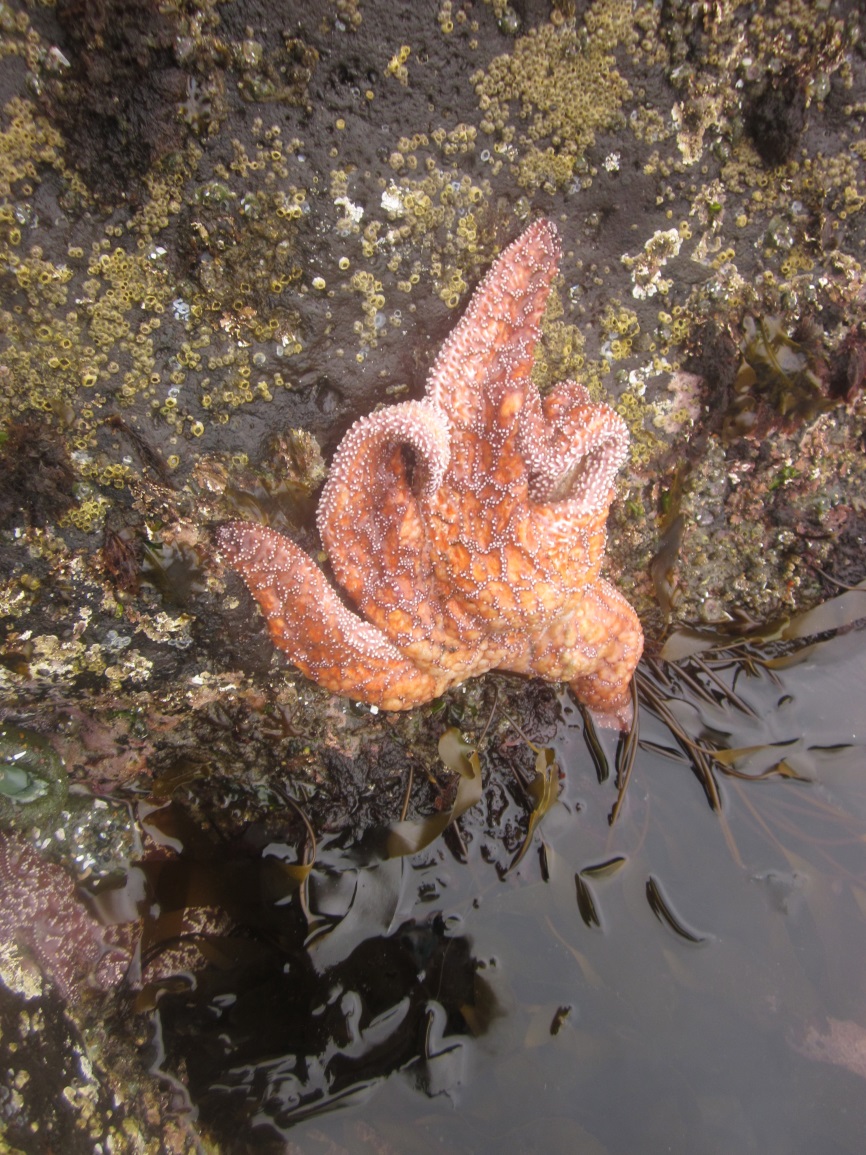


Fig D. Photos of *Pisaster ochraceus* with affliction ranking 2: Symptomatic. “Deflated” *P. ochraceus*. Such individuals have lost most or all of their coelomic fluid, and are flattened as seen in the photos.


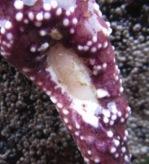

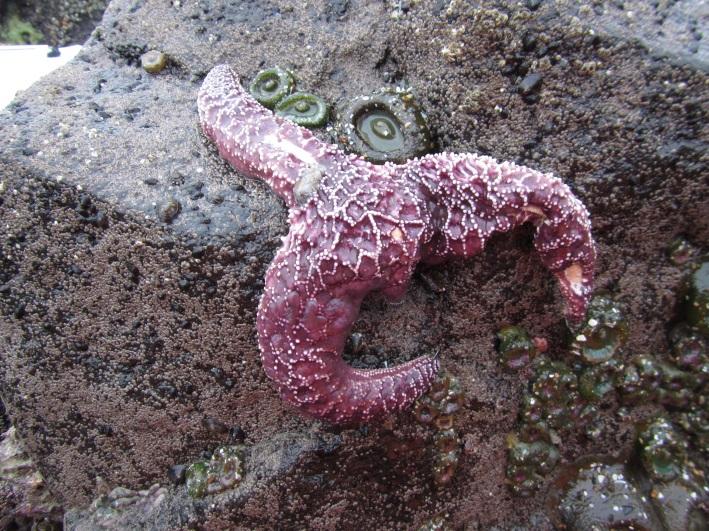


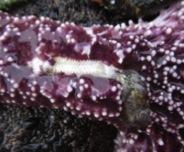


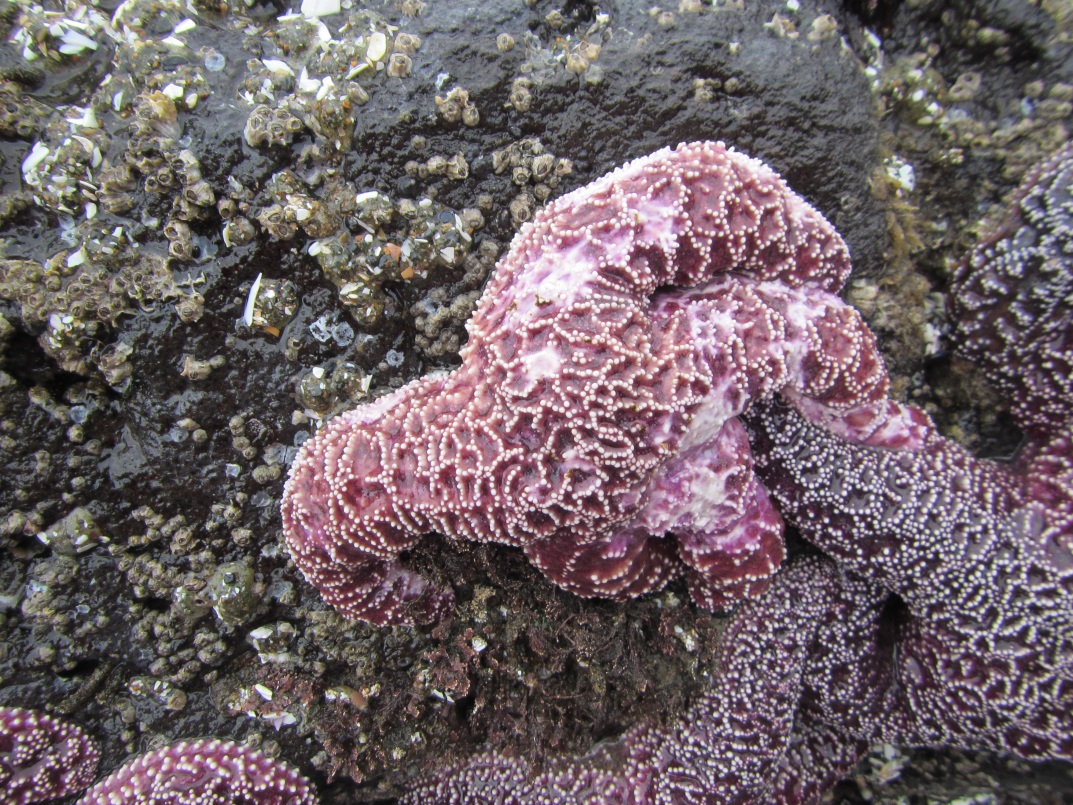


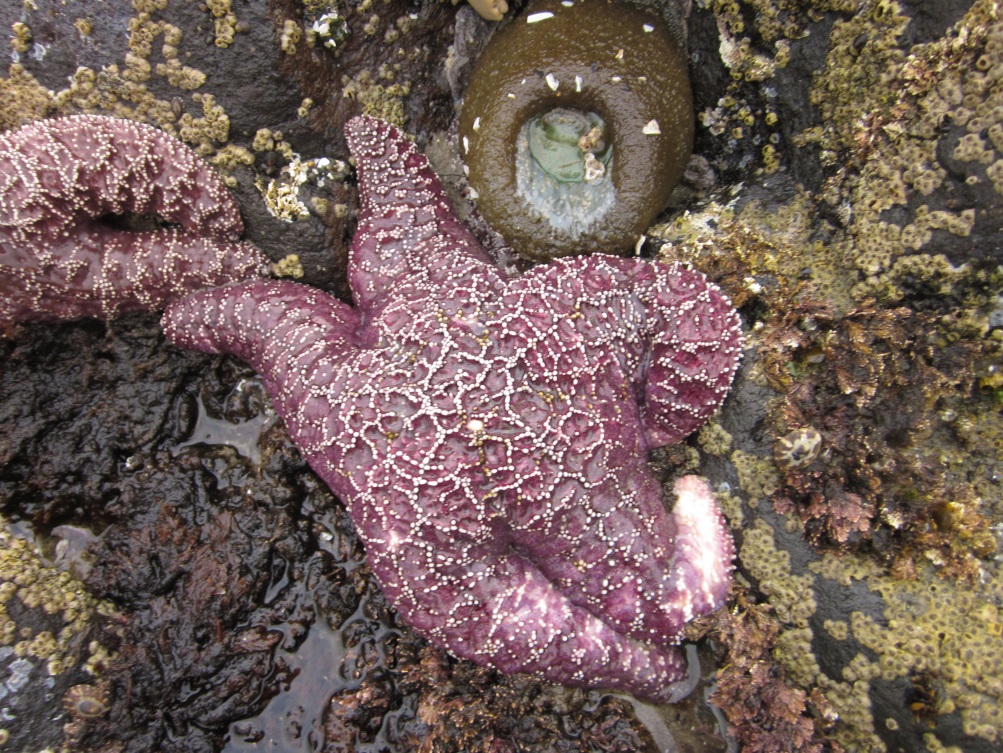


Fig E. Photos of *Pisaster ochraceus* with affliction ranking 3: Symptomatic. *P. ochraceus* with lesions. The individual in the top photo has lesions that have penetrated the body wall. The individual in the bottom two photos show early stages of multiple lesions.


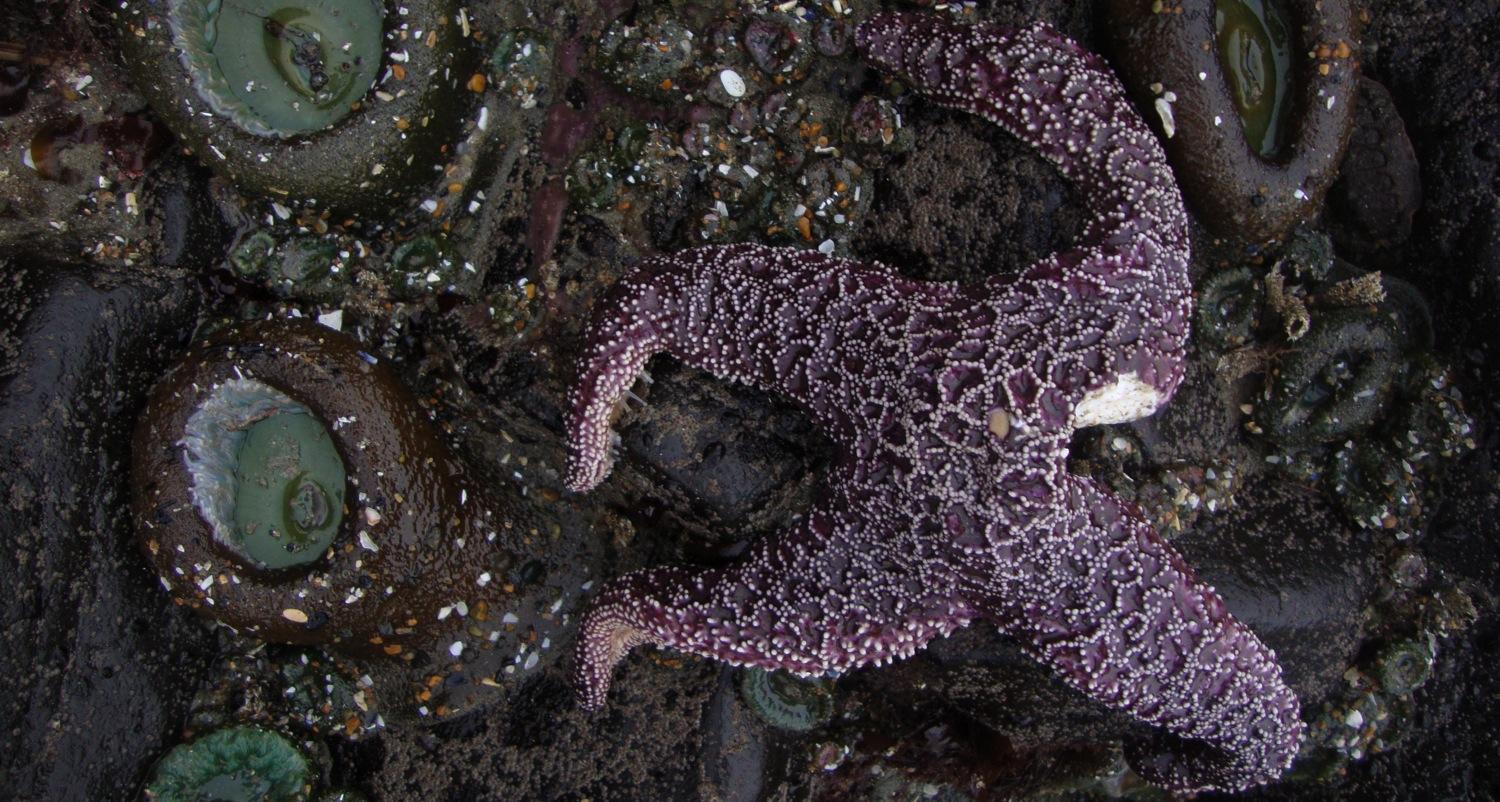

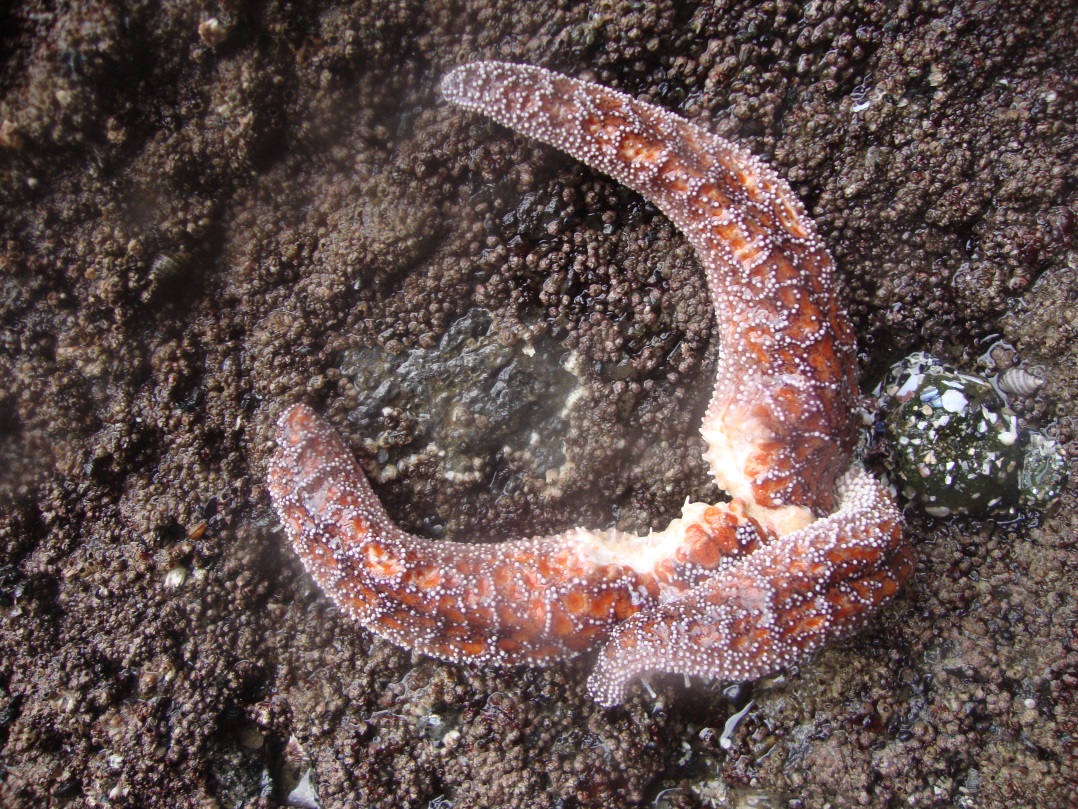

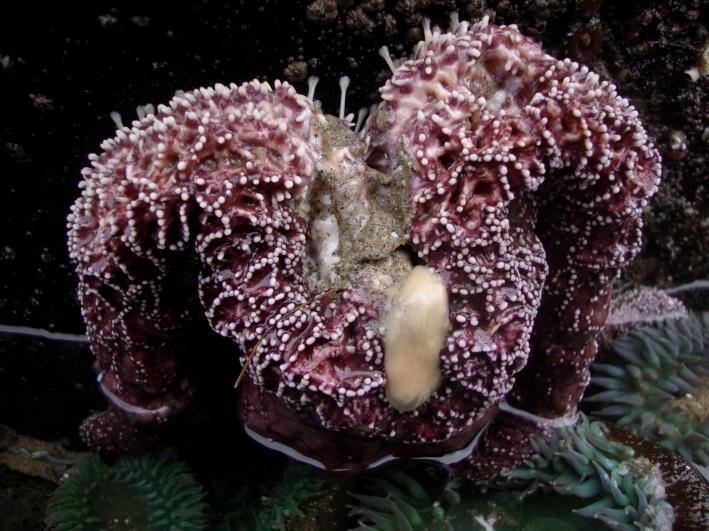


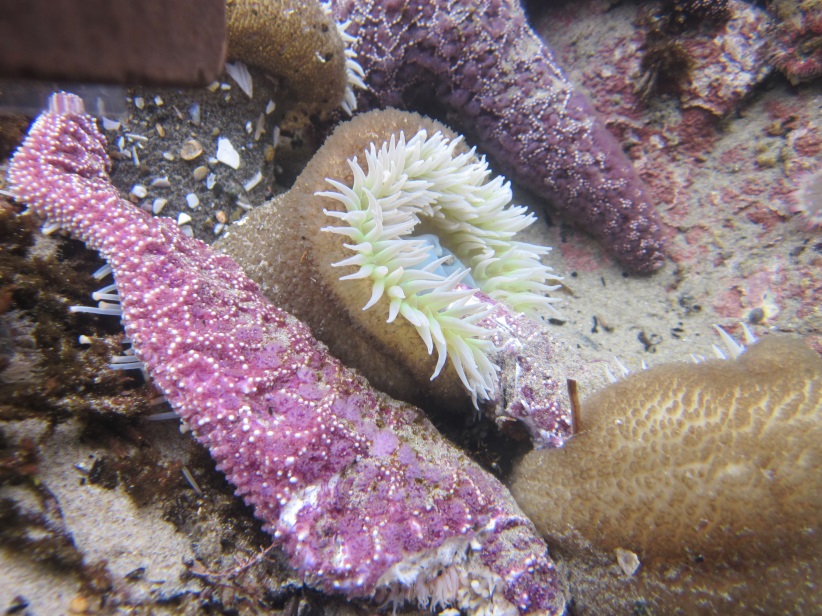


Fig F. Photos of *Pisaster ochraceus* with affliction ranking 4: Symptomatic. *P. ochraceus* with lost arms or rays. More than one arm can be lost, and sometimes just the distal portion of the arm comes off, leaving the proximal part. The bottom photo shows the sometime fate of lost arms; an anemone (*Anthopleura xanthogrammica*) is ingesting an arm in the middle of the photo. Another lost arm is right below the anemone.


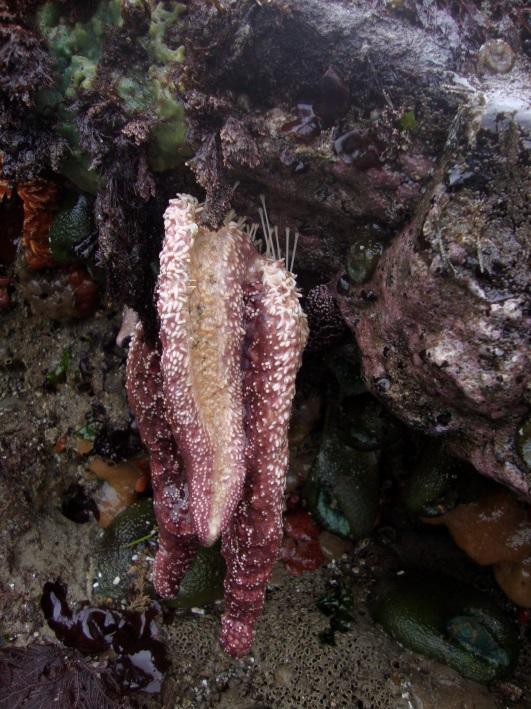

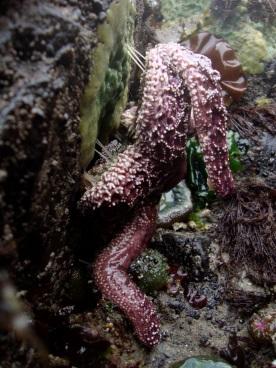


Fig G. Photos of *Pisaster ochraceus* with affliction ranking 5: Symptomatic. *P. ochraceus* losing grip on the rock. The strands attaching the animals are tube feet.


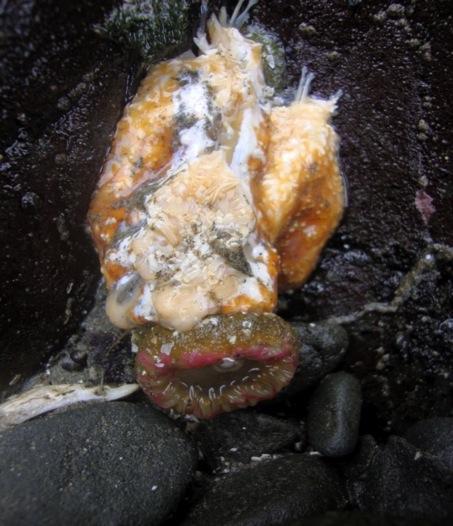


Fig H. Photos of *Pisaster ochraceus* with affliction ranking 6: Symptomatic. Final stage of disintegration, wasting, or “melting”. An anemone (*A. xanthogrammica*) is present below the pictured individual.

Fig I. Proportions of six sea star wasting disease symptoms for *P. ochraceus* at 9 sites. Data are averaged across samples taken from April to September 2014. See Methods and Appendix 1 for description of symptoms. See Fig. 1 caption for site names.

Fig J. Decline in *P. ochraceus* biomass by sites within capes, with Cape Foulweather sites on the top row (A, B), Cape Perpetua sites in the middle row (C-E), and Cape Blanco sites at the bottom (F, G). Numbers in each panel are the magnitude of biomass decline (e.g., “fold change”) and the proportion of biomass remaining in summer 2015. See Fig. 1 caption for site names.

Fig K. Cape-scale temperatures for April, May and June 2014. Data are means +1 SE of average daily temperatures for each month. Bars with different letters are different at p < 0.05 based on linear contrasts after two-way ANOVA. Gray bars are for capes (CM = Cape Meares; CMe = Cape Mendocino) or sites (SR = Seal Rock) other than the three that are focused on in the SSWD surveys and provide a wider thermal environmental context.

Fig L. Comparison of average climatology of monthly standard deviation to average monthly standard deviation in 2014 for air and water temperatures at three representative sites.

Fig M. Comparison of average climatology of maximum and minimum daily temperatures to those in 2014 for air and water temperatures at three representative sites.

Fig N. Water temperature and pH in spring and summer 2014 at Strawberry Hill. Data are from sensors deployed on nearshore moorings in 15 m depth (sensor at 4 m depth) and in the intertidal at -0.31 m MLLW.

**10:20 AM**
